# A measure of the impact of CV incompleteness on prediction error estimation with application to PCA and normalization

**DOI:** 10.1186/s12874-015-0088-9

**Published:** 2015-11-04

**Authors:** Roman Hornung, Christoph Bernau, Caroline Truntzer, Rory Wilson, Thomas Stadler, Anne-Laure Boulesteix

**Affiliations:** Department of Medical Informatics, Biometry and Epidemiology, University of Munich, Marchioninistr. 15, Munich, D-81377 Germany; Leibniz Supercomputing Center, Boltzmannstr. 1, Garching, D-85748 Germany; Clinical and Innovation Proteomic Platform, Pôle de Recherche Université de Bourgogne, 15 Bd Maréchal de Lattre de Tassigny, Dijon, F-21000 France; Department of Urology, University of Munich, Marchioninistr. 15, Munich, D-81377 Germany

**Keywords:** Cross-validation, Error estimation, Over-optimism, Practical guidelines, Supervised learning

## Abstract

**Background:**

In applications of supervised statistical learning in the biomedical field it is necessary to assess the prediction error of the respective prediction rules. Often, data preparation steps are performed on the dataset—in its entirety—before training/test set based prediction error estimation by cross-validation (CV)—an approach referred to as “incomplete CV”. Whether incomplete CV can result in an optimistically biased error estimate depends on the data preparation step under consideration. Several empirical studies have investigated the extent of bias induced by performing preliminary supervised variable selection before CV. To our knowledge, however, the potential bias induced by other data preparation steps has not yet been examined in the literature. In this paper we investigate this bias for two common data preparation steps: normalization and principal component analysis for dimension reduction of the covariate space (PCA). Furthermore we obtain preliminary results for the following steps: optimization of tuning parameters, variable filtering by variance and imputation of missing values.

**Methods:**

We devise the easily interpretable and general measure CVIIM (“CV Incompleteness Impact Measure”) to quantify the extent of bias induced by incomplete CV with respect to a data preparation step of interest. This measure can be used to determine whether a specific data preparation step should, as a general rule, be performed in each CV iteration or whether an incomplete CV procedure would be acceptable in practice. We apply CVIIM to large collections of microarray datasets to answer this question for normalization and PCA.

**Results:**

Performing normalization on the entire dataset before CV did not result in a noteworthy optimistic bias in any of the investigated cases. In contrast, when performing PCA before CV, medium to strong underestimates of the prediction error were observed in multiple settings.

**Conclusions:**

While the investigated forms of normalization can be safely performed before CV, PCA has to be performed anew in each CV split to protect against optimistic bias.

**Electronic supplementary material:**

The online version of this article (doi:10.1186/s12874-015-0088-9) contains supplementary material, which is available to authorized users.

## Background

In supervised statistical learning, it is widely recognized that prediction models should not be constructed and evaluated using the same dataset. While the training dataset is used for all steps towards obtaining the prediction rule, the test dataset is used to evaluate its prediction error and, ideally, should not be at all involved in the training phase. Cross-validation and related procedures consist of considering several divisions into training data and test data and averaging the estimated prediction errors of the respective prediction rules constructed in each iteration. In our paper we use *K*-fold cross-validation (“CV”), but all ideas and procedures can be extended to other resampling techniques used for prediction error estimation.

By “incomplete CV” [1], we are referring to CV procedures in which some analysis steps are performed beforehand using the whole dataset. With incomplete CV, at each iteration the excluded fold acting as test data may affect the derived prediction rule, since it was preliminarily used for data preparation—which contradicts the principle of test data requiring perfect separation [2]. In contrast, if all steps leading to the prediction rules are performed in each CV iteration using only the corresponding training set, the CV procedure is “full CV”.

The problems resulting from incomplete CV have been extensively studied in the literature with regard to preliminary variable selection for classification based on high-dimensional microarray data [1, 3–5]. If performed *before* splitting the dataset into *K* folds, supervised variable selection often leads to strongly downwardly biased error estimates. The now widely adopted procedure to avoid this problem consists of conducting the variable selection step in each CV iteration anew using the training dataset only [1, 3], i.e. considering it as part of the classifier construction process. Similarly, it has been suggested that parameter tuning should be performed using the training dataset only [6–8]. However, the bias resulting from incomplete CV with respect to parameter tuning has to our knowledge never been investigated in the literature.

Variable selection and parameter tuning are—by far—not the only procedures often run in practice before CV. For example, raw data from high-throughput biological experiments such as microarrays have to be normalized before so-called high-level analyses such as predictive modeling can be conducted. The selection of features which exhibit high variability across the observations is another example of a data preparation step often performed when analyzing microarray data. Further examples relevant to any type of data include imputation of missing values, dichotomization and non-linear transformations of the features. In this paper, all these procedures are designated *preparation steps* to stress that they are performed before the construction of the prediction rule. Preparation steps are not limited to these few examples. The analysis of growingly complex biomedical data (including, e.g., imaging or sequencing data) increasingly requires the use of sophisticated preprocessing steps for making raw data analysable. Note, however, that the question of the impact of CV incompleteness is not relevant to those data preparation steps which prepare the observations independently of each other, such as background correction for microarray data.

It is an open question whether preparation steps lead to underestimation of the prediction error if performed before splitting the dataset into *K* folds, as seen with variable selection. To date there seems to be no consensus on whether it is necessary to include all steps in CV: Some authors postulate that all steps are required to be included [9], which seems to be done rarely, regardless; others only suggest this procedure for variable selection [3] or more general supervised steps [10].

Practical problems which deter researchers from performing full CV are, among others, the computational effort often implied by the repetition of time-intensive preparation steps, that some preparation steps such as variable selection are sometimes conducted “in the lab” before the data are given to the statistician [11], and the lack of user-friendly implementations of addon procedures allowing the adequate preparation of the excluded fold when the preparation step has been conducted using the training folds only; see the section Addon procedures for more details on addon procedures. Another example is genotype calling in the context of genetic association studies: it is common practice to use not only the whole dataset of interest, but also further datasets, to improve genotype calling accuracy.

In the context of high-dimensional data, two further important preparation steps often performed using the whole dataset are dimension reduction procedures such as Principal Component Analysis (PCA) and normalization—for example normalization using the RMA (“Robust Multi-array Average”) method [12] for microarray gene expression data. It is not clear whether the resulting prediction error estimate is optimistically biased if one applies these two methods to the whole dataset before splitting the data into *K* folds. In an effort to answer this question we present a new measure which enables the quantification of the impact of incomplete CV with regard to steps of interest, the “CV Incompleteness Impact Measure” (CVIIM). It is based on the ratio of the CV prediction error resulting when the investigated preparation steps are applied only once using the whole dataset to the CV prediction error resulting when they are incorporated into CV. By incorporating preparation steps into CV we mean that they are performed in CV on each training dataset anew and subsequently applied to the excluded fold via so-called addon procedures.

The goal of this paper is two-fold: (i) to provide a new measure—the “CVIIM”—which is intended to be used by methodological researchers or statisticians working on statistical learning applications to determine whether a particular preparation step should—in general—be trained in each CV iteration successively or whether it can be safely performed as a preliminary step on the whole dataset without generating a relevant optimistic bias; and (ii) to apply this new measure to answer this question and provide guidelines for two important preparation steps, PCA and normalization, in the case of high-throughput molecular data.

The paper is structured as follows: the section Methods first presents the microarray gene expression datasets used in our studies, the concept of addon procedures and the two methods—normalization and PCA. Then we introduce CVIIM and briefly illustrate its use and behavior in the well-investigated case of variable selection, using four example datasets. Concluding this section we describe the designs of the studies on the impact of CV incompleteness with respect to normalization and PCA; the results of these studies are presented in the section Results. In the section Discussion we present preliminary results obtained for other data preparation steps and discuss further issues. The section Conclusions summarizes the main conclusions of the paper.

## Methods

### Data material

We used a wide range of publicly available, high-dimensional, mostly transcriptomic datasets in our real data analyses. See Table 1 for an overview. With the exception of ProstatecTranscr all datasets were downloaded from the ArrayExpress database [13] or the NCBI GEO database [14]. All datasets feature a binary target variable and are of human origin. Details on the biological background of the datasets may be obtained online via the respective accession numbers available from Table 1 and via the R scripts written for the preparation of the individual datasets for analysis. The latter are available from Additional file 1 and can be used to download and prepare the individual datasets automatically. The dataset ProstatecTranscr appeared in [15] and is available in the form of an Rda-file from Additional file 1 as well. Here we also provide R scripts for reproducing all our analyses.

**Table 1:** Overview of the datasets used in the studies on normalization and PCA. The following information is given: accession number, number of observations, number of variables, proportion of observations in the smaller class, data type

Study	Label/	Num. of	Num. of	Prop. smaller	Data type	ID
	acc. number	observ.	variables	class		
Normalization	E-GEOD-10320	100	22283	0.42	transcription	1
Normalization	E-GEOD-47552	74	32321	0.45	transcription	2
Normalization	E-GEOD-25639	57	54675	0.46	transcription	3
Normalization	E-GEOD-29044	54	54675	0.41	transcription	4
Normalization	E-MTAB-57	47	22283	0.47	transcription	5
Normalization	E-GEOD-19722	46	54675	0.39	transcription	6
Normalization	E-MEXP-3756	40	54675	0.50	transcription	7
Normalization	E-GEOD-34465	26	32321	0.35	transcription	8
Normalization	E-GEOD-30174	20	54675	0.50	transcription	9
Normalization	E-GEOD-39683	20	32321	0.40	transcription	10
Normalization	E-GEOD-40744	20	20706	0.50	transcription	11
Normalization	E-GEOD-46053	20	54675	0.40	transcription	12
PCA	E-GEOD-37582	121	48766	0.39	transcription	13
PCA	ProstatecTranscr	102	12625	0.49	transcription	14
PCA	GSE20189	100	22277	0.49	transcription	15
PCA	E-GEOD-57285	77	27578	0.45	DNA methyl.	16
PCA	E-GEOD-48153	71	23232	0.48	proteomic	17
PCA	E-GEOD-42826	68	47323	0.24	transcription	18
PCA	E-GEOD-31629	62	13737	0.35	transcription	19
PCA	E-GEOD-33615	60	45015	0.35	transcription	20
PCA	E-GEOD-39046	57	392	0.47	transcription	21
PCA	E-GEOD-32393	56	27578	0.41	DNA methyl.	22
PCA	E-GEOD-42830	55	47323	0.31	transcription	23
PCA	E-GEOD-39345	52	22184	0.38	transcription	24
PCA	GSE33205	50	22011	0.50	transcription	25
PCA	E-GEOD-36769	50	54675	0.28	transcription	26
PCA	E-GEOD-43329	48	887	0.40	transcription	27
PCA	E-GEOD-42042	47	27578	0.49	DNA methyl.	28
PCA	E-GEOD-25609	41	1145	0.49	transcription	29
PCA	GSE37356	36	47231	0.44	transcription	30
PCA	E-GEOD-49641	36	33297	0.50	transcription	31
PCA	E-GEOD-37965	30	485563	0.50	DNA methyl.	32

ArrayExpress accession numbers have the prefix E-GEOD-, NCBI GEO accession numbers have the prefix GSE

In the search for suitable datasets we excluded those which featured a strong class imbalance or which would have been difficult to handle from a computational point of view.

### Addon procedures

In this section we give a brief overview of the crucial concept of addon procedures. When a data preparation step has been conducted on the training data only, the test data must be prepared equivalently: to not do so might render the test data nonsensical with regard to—or even incompatible with—the prediction rule derived on the training data. A naive but straightforward procedure for steps which do not involve the response variable (“unsupervised” steps) such as normalization (see the section (Addon) normalization), is to prepare the test data completely independently, i.e. without using any information from the preparation of the training data. For the prediction of external data, such a separate data preparation procedure may be suitable in some situations, for example when the external data behaves very differently from the training data: by a separate processing the data preparation procedure can adjust itself to the peculiarities of the external data; see e.g. [16]. However, in general this approach may lead to a higher prediction error in the case of small test datasets because of the larger variance of the output of preparation steps. Test datasets of size 1 (corresponding to, say, patients examined one at a time) are an extreme case where this approach is completely infeasible. Moreover, for some preparation steps such as variable filtering by variance this naive approach cannot be applied since it would lead to the selection of different variables in the training and test datasets and thus make the application of the prediction rule impossible.

Another straightforward idea is to “train” the preparation step on the training data and to use the output of the preparation step to prepare the test data. We refer to such a procedure as an “addon procedure”. This term was originally introduced in the specific case of normalization for microarray data [17] but is employed here for all types of data preparation steps. We give the following definition: an addon procedure for a preliminary step is a procedure which prepares an observation in the test data precisely as it would prepare a corresponding observation in the training data, using empirical information derived exclusively from the training data. Note that by “performing” a preliminary step we mean more precisely: 1) conduct the preparation step on the considered data; 2) store all information necessary for addon preparation of new observations. Addon procedures are trivial in some cases, for instance that for dichotomization according to cutpoints determined from the training data (one simply uses the training-data-derived cutpoint to dichotomize the test data) or in the case of variable selection (selecting precisely those variables in the test data which were selected based on the training data). In other cases, like normalization of microarray data or imputation of missing values, however, this task is more complex.

### (Addon) normalization

Normalization of microarray data is, roughly speaking, the transformation of the data in such a way as to eliminate—or reduce—systematic differences between observations which are unrelated to biological differences. In this paper we consider two different methods of microarray data normalization: 1) RMA; and 2) RMA where the quantile-normalization step is expanded by VSN (“Variance Stabilization Normalization”) [18] without calibration (RMAglobalVSN) [19]. VSN transforms the gene expression values in such a way that the variance of the differences between values of different observations is approximately constant along the whole intensity range. For the quantile normalization step of RMA we use the addon procedure provided by Kostka and Spang [17] whenever full CV is performed. Here, the quantiles of the test observations are replaced by the quantiles of the training observations after quantile normalization of the latter. Since background correction and summarization are performed on an array-by-array basis, no addon strategies are necessary for these procedures. In the vignette of the Bioconductor package vsn, Huber [19] presents a version of variance stabilization in which no calibration is performed, i.e. only a global variance stabilization transformation is conducted. In contrast to standard VSN this procedure does not involve any observation-specific parameters, so it is possible to determine an addon procedure: the global VSN parameters estimated on the training data are used to transform the test data.

### (Addon) principal component analysis (PCA)

PCA is an unsupervised dimension reduction method commonly used in the context of high-dimensional data analysis. The principal components are calculated using a singular value decomposition of the centered data matrix. The addon procedure works as follows: 1) Center the values of each variable by subtracting the corresponding variable mean estimated from the training data; 2) Multiply the matrix resulting from 1) by the PCA loading matrix derived from the training data to obtain the principal components. The principal components with highest variance can be viewed as summarizing the data in fewer dimensions, and are often used in practice for graphical representation of the data. In the context of classification using high-dimensional data, it is common to fit a prediction rule with a prediction method such as Discriminant Analysis using principal components as predictors instead of the original variables [20].

### The cross-validation incompleteness impact measure (CVIIM)

In the following we present CVIIM, our new measure for the extent of bias induced by incomplete CV with respect to a data preparation step of interest. Let ***s*** be the available dataset from which a prediction rule is to be derived. ***s*** is assumed to be an *i.i.d.* sample of size *n* with observations drawn from the distribution *P*, where *P* is the joint distribution of predictors and response variable. Note that the assumption of *i.i.d.* observations made here is owing to the fact that throughout this paper we are concerned with cross-validation, i.e. dataset internal validation. With external validation this assumption is generally not appropriate. Further, let *e*_*full*,*K*_(***s***) be the prediction error estimated by full *K*-fold CV, i.e. when all steps leading to the prediction rule, including data preparation steps, are performed at each CV iteration anew based only on the training dataset. Similarly let *e*_*incompl*,*K*_(***s***) be the prediction error estimated by incomplete *K*-fold CV, i.e. when the data preparation step(s) of interest is performed before CV, using the whole dataset. For simplicity of notation, we additionally assume that *e*_*full,K*_(***s***) and *e*_*incompl*,*K*_(***s***) are obtained by averaging over a large number of CV runs, i.e. over a large number of random partitions, and can thus be treated as deterministic.

For ***S***∼*P*^*n*^, our new measure “CVIIM”, “Cross-Validation Incompleteness Impact Measure”, is defined as:

(1) $$ \fontsize{8.3}{8} {}\text{CVIIM}_{P, n, K} \! :=\! \left\{\! \begin{array}{ll} 1- \frac{\text{E}[e_{incompl, K}(\boldsymbol{S})]}{\text{E}[e_{full, K}(\boldsymbol{S})]} &\! \text{if} \,\, \text{E}[\!e_{incompl, K}(\boldsymbol{S})] < \text{E}[\!e_{full, K}(\boldsymbol{S})] \\ &\! \text{and} \ \text{E}[\!e_{full, K}(\boldsymbol{S})] > 0 \\ \\ 0 &\! \text{otherwise.} \end{array}\right.  $$

Note that we defined CVIIM*P,n,K* as a theoretical quantity, not calculable, but estimable from real data. It is simply estimated by replacing the expected CV errors by their empirical counterparts *e*_*incompl*,*K*_(***s***) and *e*_*full*,*K*_(***s***):

(2) $$ \fontsize{8.3}{8} {}\text{CVIIM}_{\boldsymbol{s}, n, K} \; := \; \left\{ \begin{array}{ll} 1 \; - \; \frac{e_{incompl, K}(\boldsymbol{s})}{e_{full, K}(\boldsymbol{s})} & \text{if} \;\;e_{incompl, K}(\boldsymbol{s}) \, < \, e_{full, K}(\boldsymbol{s}) \\ & \text{and} \ e_{full, K}(\boldsymbol{s}) \, > \, 0 \\ \\ 0 & \text{otherwise.} \end{array}\right.  $$

Clearly, CVIIM*P,n,K*∈[0,1]. The same holds for the estimator CVIIM***s***,*n,K*. CVIIM is based on the ratio of the incomplete CV error to the full CV error, which is more revealing than their difference as a measure of the impact of CV incompleteness. Indeed, the latter would strongly depend on the value of the error (large error values leading to large differences), as suggested by the results shown in the section Alternative measures of CV incompleteness and by our simulation presented in the section Simulation study and in Appendix A (Additional file 2). Truncation at 0 prevents CVIIM from being negative in the unlikely case that incomplete CV error is larger than the full CV error. A large value of CVIIM indicates that CV incompleteness results in a large underestimation of prediction error.

The discrepancy between *e*_*incompl*,*K*_(***s***) and *e*_*full*,*K*_(***s***) depends on how strongly the specific preliminary step conducted on the whole dataset increases the homogeneity of the covariate values across observations and (for supervised preparation steps) the empirical association between the covariate values and the values of the target variable.

With the interpretation of CVIIM***s***,*n,K* in mind and based on real data results and expectations regarding the impact of specific data preparation steps, we define the following tentative rules of thumb for categorizing the computed values in terms of the impact of CV incompleteness with regard to the considered step(s): [0,0.02]∼ no influence, ]0.02,0.1]∼ weak, ]0.1,0.2]∼ medium, ]0.2,0.4]∼ strong, ]0.4,1]∼ very strong.

We outline an artificial example to demonstrate, step by step, a possible application of CVIIM*P,n,K*. We are interested in measuring the extent of overoptimism connected with performing the quantile normalization step of RMA before CV in gene expression based classification. Suppose we have a dataset with gene expression measurements from 32 patients suffering from breast cancer and from 22 disease-free patients. Per patient we have measurements of the expression of 54,675 genes. As classification method we use Nearest Shrunken Centroids (NSC). The error *e*_*incompl*,5_(***s***), as estimated by incomplete 5-fold CV, is computed by conducting the RMA normalization beforehand on the whole dataset and performing 5-fold CV on the normalized dataset. In this procedure only the fitting of NSC is repeated in each CV iteration on the training datasets. The CV is repeated 300 times to obtain more stable results. The full CV error *e*_*full*,5_(***s***) is computed by performing a 5-fold CV in which the quantile normalization step of RMA (as well as the fitting of the NSC) is re-performed in each CV iteration on the respective training set, with addon normalization of the corresponding test set through the addon procedure by Kostka and Spang [17]. This procedure is again repeated 300 times. Suppose we were to obtain *e*_*incompl*,5_(***s***)=0.15 and *e*_*full*,5_(***s***)=0.1503. Then, CVIIM***s***,*n,K*=1−0.15/0.1503∼0.002. According to our rules of thumb this would correspond to no influence on the estimated error.

This result obtained for a specific dataset and specific classifier, however, may not be representative of all datasets and classifiers in the field of gene expression based classification. Extending this example, we point out that it is necessary to study several datasets and several analysis settings representative of the considered field in order to formulate recommendations regarding incomplete CV for a particular step. Alternatively, specific guidelines could be formulated for particular settings and data types within the considered field; however, this might easily lead to overly complicated guidelines.

For a formal introduction to the concepts involved in this section such as prediction rules, prediction error, and its estimation via full and incomplete CV the interested reader may consult Appendices B.1 and B.2 (Additional file 2).

### Global CVIIM

As outlined above, the value of CVIIM***s***,*n,K* obviously depends on the specific dataset. For a general assessment of the bias attributable to a specific step we need a more global measure summarizing the results obtained on several datasets. To this end we define the *global CVIIM* as the quantity resulting when replacing E[*e*_*incompl*,*K*_(***S***)] and E[*e*_*full*,*K*_(***S***)] in (1) by, roughly speaking, their means over the universe of datasets from the area of interest (see [21] for a more formal description of this concept in another context). Consider the following example: at this time the standard approach in microarray data analysis is to perform quantile normalization of RMA on the whole dataset before performing CV. Suppose that the prediction error is, on average, 0.2 over all datasets from the area of interest, but if full CV were performed with respect to quantile normalization it would equal 0.201. Then the global CVIIM in this scenario would be 1−0.2/0.201∼0.005, a negligibly weak overall bias.

To estimate the global CVIIM we suggest the plug-in estimator obtained by replacing *e*_*incompl*,*K*_(***s***) and *e*_*full*,*K*_(***s***) in Eq. (2) by the averages of their values obtained on several datasets from the considered area of application:

$$\begin{aligned} &\text{CVIIMglobal}_{\boldsymbol{s}^{(1)},\dots,\boldsymbol{s}^{(L)};K} \\:= &\left\{\!\! \begin{array}{ll} 1 - \frac{\frac{1}{L}\sum_{l=1}^{L} e_{incompl, K}(\boldsymbol{s}^{(l)})}{\frac{1}{L}\sum_{l=1}^{L} e_{full, K}(\boldsymbol{s}^{(l)})} & \; \text{if} \;\;\frac{1}{L}\sum_{l=1}^{L} e_{incompl, K}(\boldsymbol{s}^{(l)}) \, <\\ \; \, &\frac{1}{L}\sum_{l=1}^{L} e_{full, K}(\boldsymbol{s}^{(l)}) \;\\ & \text{and } \frac{1}{L}\sum_{l=1}^{L} e_{full, K}(\boldsymbol{s}^{(l)}) \, > \, 0 \\ \\ 0 & \text{otherwise,} \end{array}\right. \end{aligned} $$

where ***s***^(1)^,…,***s***^(*L*)^ are the datasets used. Note that this estimator is not strongly affected by individual extreme CVIIM estimates, which can occur in the case of very small values of E[*e*_*full*,*K*_(***S***)]. For a detailed discussion of this phenomenon, see Appendix B.3 (Additional file 2).

### Illustration

To give a first illustration of the application of CVIIM as a proof of concept, we apply it to supervised variable selection, which is expected to yield high CVIIM values. We use the datasets ProstatecTranscr, GSE33205, GSE20189 and GSE37356, which are also considered in the PCA study; see Table 1.

For each variable a two-sample t-test is conducted to test the equality of the means of the two groups. The variables with the smallest *p*-values are selected. Because it is expected that the result substantially depends on the number of selected variables, the analysis is repeated for different numbers of variables: 5, 10, 20 and half of the total number *p* of variables. After selecting 5, 10 and 20 variables we use LDA as a classification method. When selecting half of the variables LDA cannot be applied, because the involved empirical covariance matrices are not well-behaved in general when the number of variables is higher than the number of observations. In this case, we use Diagonal Linear Discriminant Analysis, i.e. LDA under the simplifying assumption that within the two classes the variables are independent; see Hastie et al. [10].

In all analyses performed in the paper, *e*_*incompl*,*K*_(***s***) and *e*_*full*,*K*_(***s***) are obtained by averaging the results from *B*=300 runs of *K*-fold CV, where *K* takes the values 3, 5 and 10 successively.

**Fig. 1:**
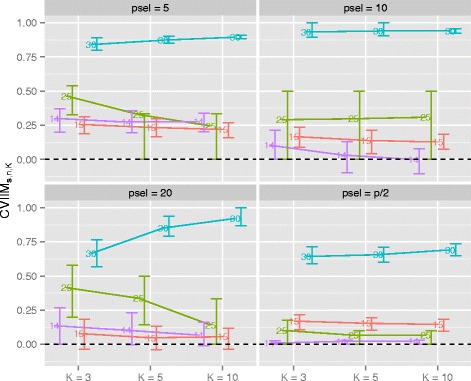
CVIIM***s***,*n,K*-values from variable selection study. The numbers distinguish the datasets. psel denotes the number of selected variables

The CVIIM***s***,*n,K*-values obtained for all settings are displayed in Fig. 1. In the plots the error bars represent the 25 %- and 75 %-quartiles (computed over the *B*=300 iterations) of the iterationwise non-truncated incompleteness measure estimates (INIMEs) CVIIM***s***,*n,K,b*:=1−*e*_*incompl*,*K*_(***s***)_*b*_/*e*_*full*,*K*_(***s***)_*b*_, where the index *b* indicates that these errors are obtained for run *b* (with *b*=1,…,*B*). It is important to note that the error bars should be used for comparisons between each other only, since their absolute lengths have no relevant interpretation. Note that due to the unboundedness of the INIMEs the error bars—as opposed to the CVIIM***s***,*n,K*-values—are not bound by zero.

While CVIIM***s***,*n,K* is especially large for small numbers of selected variables, relatively large values are also observed when half of the variables are selected (with the exception of the dataset with the fewest variables). Although the differences in CVIIM***s***,*n,K* for the selection of 5, 10 and 20 variables are not large, the estimates of the global CVIIM given in Table 2 indicate that the bias induced by incomplete CV tends to decrease with an increasing number of selected variables. Dataset 30 stands out through its noticeably larger CVIIM***s***,*n,K*-values in all plots. This dataset comprises only 36 observations but 47,231 variables (see Table 1), which may at least partly explain the larger values. Extreme values above 0.9, however, are surprising.

**Table 2:** Estimates of global CVIIM from the variable selection study

Number of sel.	*K*=3	*K*=5	*K*=10
variables			
5	0.5777	0.5927	0.6126
10	0.5557	0.5617	0.5505
20	0.3971	0.4706	0.4511
p/2	0.2720	0.2702	0.2824

In this illustrative analysis, through our new measure CVIIM we have confirmed the conclusion previously obtained in the literature: performing supervised variable selection before CV leads to a strong bias of the resulting error estimate.

### Study design

The investigation of normalization is based on the first 12 microarray datasets listed in Table 1. We use the two variants of normalization described in the section (Addon) normalization. Two different classification methods are used successively to derive prediction rules: NSC and Linear Discriminant Analysis performed on Partial Least Squares components (PLS-LDA). For NSC the shrinkage intensity *Δ* is chosen from the grid {0.05,0.1,0.25,0.5,1,1.5} and for PLS-LDA the number of components *n*_*comp*_ is chosen from the grid {1,2,…,10}. Parameter choice is done in the following way. For each considered training dataset, we perform 3-fold internal CV for each candidate parameter value from the grid. The candidate parameter value yielding the smallest 3-fold CV error is selected.

The study on PCA is based on the last 20 microarray datasets listed in Table 1. The constructed principal components are used as predictors in Linear Discriminant Analysis (LDA) and Random Forest (RF), successively. For RF, the crucial parameter *mtry*, denoting the number of predictors considered as candidates in the splits of the trees, is chosen by 3-fold internal CV from the grid {1,2,3,5,10}. Since the results can be assumed to strongly depend on the number of principal components used as predictors, we repeat the analyses for four different numbers: 2, 5, 10 and 15.

## Results

### Normalization

**Fig. 2:**
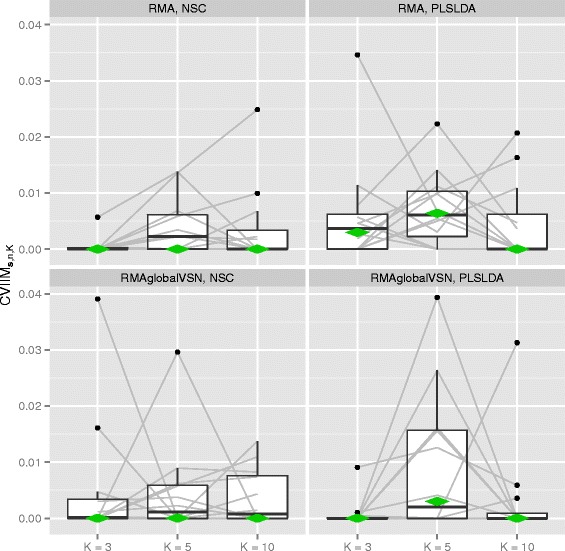
CVIIM*s,n,K*-values from normalization study. The grey lines connect the values corresponding to the same datasets. The diamonds depict the estimates of global CVIIM

Figure 2 depicts the CVIIM***s***,*n,K*-values from the normalization study together with the estimates of global CVIIM. The latter are also given in Table 3. For both normalization approaches we observe very small CVIIM***s***,*n,K*-values for all datasets and both classifiers. In the majority of cases the measure estimates suggest no bias resulting from incomplete CV for normalization as defined by our rule of thumb. The global CVIIM estimates seem to confirm that in general there is no bias. We obtain slightly higher values for PLS-LDA than for NSC, but the difference is not noteworthy.

**Table 3:** Estimates of global CVIIM from the normalization study

Normalization	Classification	*K*=3	*K*=5	*K*=10
method	method			
RMA	NSC	0.0000	0.0000	0.0000
	PLS-LDA	0.0030	0.0064	0.0000
RMAglobalVSN	NSC	0.0000	< 0.0001	0.0000
	PLS-LDA	0.0000	0.0030	0.0000

For the individual datasets there is no visible dependency of the measure estimates on *K*, although in general we expect a negative dependency; see the section Further issues for a discussion of this topic. The fact that we do not observe such a decrease with *K* for normalization can likely be explained by the small values of the estimates: *e*_*incompl*,*K*_(***s***) and *e*_*full*,*K*_(***s***) are very similar here. Therefore the non-systematic fluctuations across the different *K*-values are attributable to small—probably random—fluctuations of *e*_*incompl*,*K*_(***s***) and *e*_*full*,*K*_(***s***) over *K*, which could overshadow a potential dependency on *K*.

In contrast to the section Illustration, we do not present iteration-based error bars for the individual CVIIM***s***,*n,K*-values here. When depicting the results of a study with a larger number of datasets individual error bars make the corresponding plots increasingly unclear. Instead in this situation we focus on the distribution of the CVIIM***s***,*n,K*-values across datasets—the results over individual datasets are less important. Nevertheless extreme individual results should be examined more closely.

Given the small CVIIM estimates we conclude that RMA and RMA with global VSN can be safely performed before CV without inducing a relevant bias in the resulting error estimate.

### Principal component analysis

**Fig. 3:**
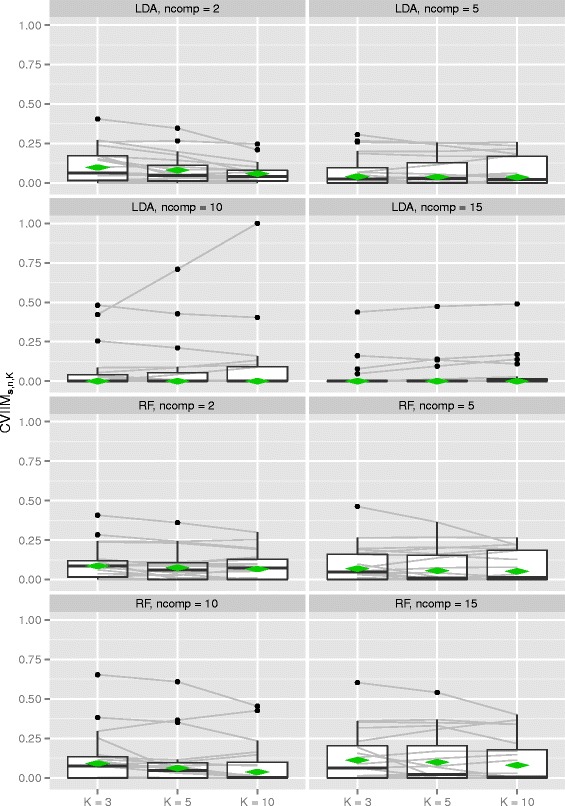
CVIIM*s,n,K*-values from PCA study. The grey lines connect the values corresponding to the same datasets. The diamonds depict the estimates of global CVIIM

Figure 3 and Table 4 show the results of the PCA study. Note that the scale of Fig. 3 is much larger than that of the corresponding plot for normalization (Fig. 2). Globally the results suggest a weak but existent underestimation of the true error E[*e*_*full*,*K*_(***S***)] by performing PCA before CV. Exceptions are LDA in those instances where the number of components is greater than five, where zero values of the global CVIIM-estimates are obtained.

**Table 4:** Estimates of global CVIIM from the PCA study

Classification	Number of	*K*=3	*K*=5	*K*=10
method	components			
LDA	2	0.0974	0.0805	0.0582
	5	0.0397	0.0371	0.0354
	10	0.0000	0.0000	0.0000
	15	0.0000	0.0000	0.0000
RF	2	0.0855	0.0747	0.0659
	5	0.0686	0.0558	0.0516
	10	0.0907	0.0613	0.0368
	15	0.1117	0.0988	0.0794

For LDA the impact of incomplete CV seems to diminish with an increasing number of components in PCA. The global CVIIM-estimates are in general larger for RF than for LDA. While the overall effects of performing PCA before CV seem to be weak, Fig. 3 reveals that there are several settings in which the CVIIM-estimates suggest a strong bias—according to our rules of thumb—for a non-neglible number of datasets. Therefore, these results strongly suggest the use of full CV over incomplete CV with respect to PCA.

A closer look at Table 4 reveals that, in general, the global CVIIM-estimates decrease with increasing value of *K* (for all settings with non-zero values). For example, this decrease is noticeable for LDA with *ncomp*=2 and RF with *ncomp*=10. This suggests that the estimates of global CVIIM are overly high in these cases, due to the greater upward bias of *e*_*full*,*K*_(***s***) compared to *e*_*incompl*,*K*_(***s***) as detailed in the section Further issues. The global CVIIM-estimates depend on the means in the *e*_*full*,*K*_(***s***)- and the *e*_*incompl*,*K*_(***s***)-values calculated over the included datasets. The decrease with larger values of *K* is induced by the mean of the *e*_*full*,*K*_(***s***)-values becoming more similar to the mean of the *e*_*incompl*,*K*_(***s***)-values with increasing value of *K*. For most settings we do not observe a substantial decrease of the global CVIIM-estimates. This suggests that the two cases for which the decrease with *K* was strong are connected to aberrant results for individual datasets, which was confirmed by more closely inspecting the individual values obtained for each setting and each dataset.

Motivated by this observation we performed a simple type of sensitivity analysis. First for each of the two settings we left out the dataset which displayed the largest difference between *e*_*full*,3_(***s***) and *e*_*full*,10_(***s***) and re-estimated the global CVIIM-values. For the LDA with *ncomp*=2 the results were 0.0812 (*K*=3), 0.0681 (*K*=5) and 0.0524 (*K*=10), and for RF with *ncomp*=10 we obtained 0.0590 (*K*=3), 0.0351 (*K*=5) and 0.0222 (*K*=10). The values are obviously more similar across the three different *K*-values for both settings compared to the results obtained when using all 20 datasets; see again Table 4. This is especially noticeable in the case of the values for *K*=5 and *K*=10 in “LDA with *ncomp*=2”. Nevertheless there are still significant differences. Therefore, as a second step we repeated the same procedure, this time however leaving out the three datasets with the largest differences between *e*_*full*,3_(***s***) and *e*_*full*,10_(***s***). The results were: 0.0676 (*K*=3), 0.0575 (*K*=5) and 0.0499 (*K*=10) for LDA with *ncomp*=2, and 0.0067 (*K*=3), 0.0000 (*K*=5) and 0.0000 (*K*=10) for RF with *ncomp*=10. For the former setting the similarity across *K*-values has obviously increased, while at the same time the sizes of the values have not decreased strongly. The (almost) zero-values for the second setting are quite striking given that we observed values as high as 0.0907 for *K*=3 when using all 20 datasets. We also performed the same analysis for all other settings (results not shown): the global CVIIM-estimates in these settings tended to be more robust to the removal of datasets than the ones of the settings presented here. These results—especially those obtained for the setting “RF with *ncomp*=10”—illustrate that a strong decrease in the global CVIIM-estimates with increasing value of *K* should be interpreted with caution. We recommend performing sensitivity analysis in the form of the one conducted here in such cases.

## Discussion

In this section we first discuss possible alternative measures of CV incompleteness—using the PCA example—and why we deem them less appropriate than our measure CVIIM. Then we present as an outlook some preliminary results obtained for further data preparation steps beyond normalization and PCA. Finally, we discuss various further issues related to CVIIM.

### Alternative measures of CV incompleteness

**Fig. 4:**
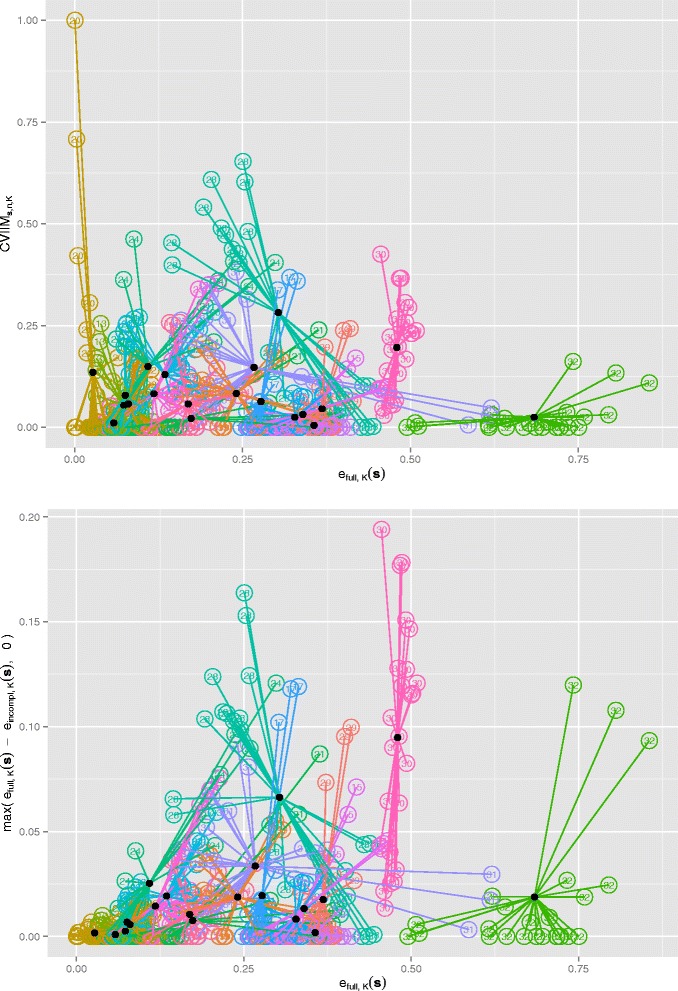
Dependency on CV errors in PCA study. *Upper panel*: CVIIM*s,n,K*-values versus *e*_*full*,*K*_(***s***)-values for all settings; *Lower panel*: Zero-truncated differences of *e*_*full*,*K*_(***s***)- and *e*_*incompl*,*K*_(***s***)-values versus *e*_*full*,*K*_(***s***)-values for all settings. The colors and numbers distinguish the different datasets. The filled black circles depict the respective means over the results of all settings obtained on the specific datasets

An important question with respect to the definition of CVIIM is whether it depends on E[*e*_*full*,*K*_(***S***)]. Such a dependence is not desirable, since CVIIM should not be a measure of the error but of the impact of CV incompleteness. To investigate this in the context of the PCA study, we plot CVIIM***s****,n,K* against *e*_*full*,*K*_(***s***) in the upper panel of Fig. 4, where the different analysis settings for a given dataset are represented using the same colour and number, and the mean of each dataset is displayed as a black point. This plot suggests no relevant dependency of CVIIM***s***,*n,K* on the full CV error *e*_*full*,*K*_(***s***). For two of the smallest errors we observe extreme CVIIM-estimates, resulting from random fluctuations in the error estimates as discussed in Appendix B.3 (Additional file 2). However, this problem—concerning only two values out of 480 error values in total—seems to be negligible. The lower panel of Fig. 4 displays the zero-truncated difference between *e*_*full*,*K*_(***s***) and *e*_*incompl*,*K*_(***s***) against *e*_*full*,*K*_(***s***). This plot clearly suggests a comparatively strong dependence of the estimates of this measure on the full CV error—as also observed in the results obtained in the simulation study presented in Appendix A (Additional file 2)—and thus provides evidence supporting the use of a ratio-based measure rather than a difference-based measure. Analogous plots give a very similar picture in the case of normalization; see Figure S6 in Appendix C (Additional file 2).

**Fig. 5:**
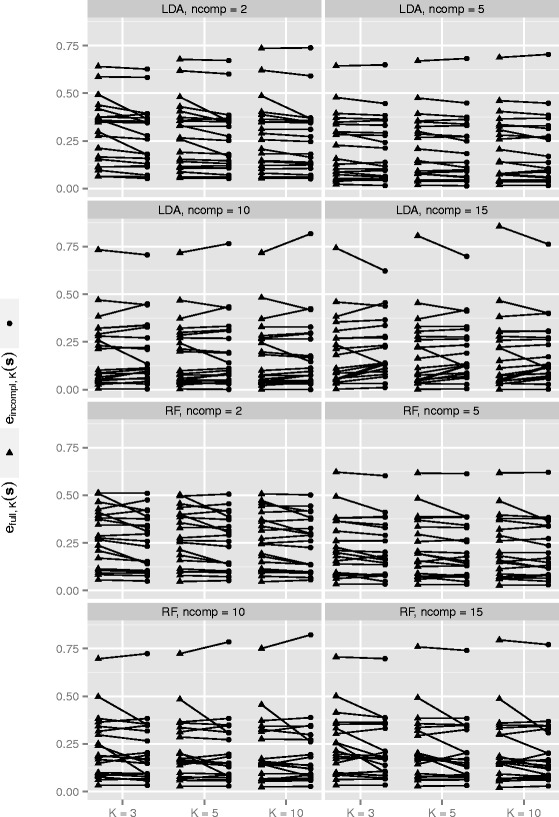
Errors in PCA study. *e*_*full*,*K*_(***s***)- and *e*_*incompl*,*K*_(***s***)-values for all datasets and settings from the PCA study

An obvious, but less insightful, way of visualizing the impact of CV incompleteness, is to simply plot *e*_*full*,*K*_(***s***) and *e*_*incompl*,*K*_(***s***) for the individual datasets. Figure 5 shows such a plot for the PCA study. Without closer inspection we observe that in some cases *e*_*incompl*,*K*_(***s***) is considerably smaller than *e*_*full*,*K*_(***s***), indicating the strong bias already suggested by the CVIIM***s***,*n,K*-values.

However, this visualization has two crucial disadvantages. Firstly, in contrast to the plot of the CVIIM-estimates, it does not show values which allow immediate interpretation of the extent of overoptimism for the individual datasets. Secondly, it draws attention to the different sizes of the errors across individual datasets rather than highlighting the discrepancies between the *e*_*full*,*K*_(***s***)- and *e*_*incompl*,*K*_(***s***)-values, which should be the actual focus of interest.

### Outlook: other preparation steps

We performed additional analyses for further data preparation steps, although with fewer datasets and fewer analysis settings than in the studies for normalization and PCA. These preparation steps were: optimization of tuning parameters, variable filtering by variance and imputation of missing values. See Appendix D (Additional file 2) for the study designs and detailed results. Here, in general, optimization of tuning parameters was connected with weak, but non-negligible optimistic biases. For variable filtering by variance and imputation of missing values the bias was negligible. Note that, due to the limited number of datasets and analysis settings, the results should not be over-interpreted. Further validation is required before practical guidelines can be formulated with respect to these preparation steps.

### Simulation study

In addition to the real data studies presented above, we also conducted a simulation study to investigate general statistical properties of CVIIM***s***,*n,K*. As the preparation step we used supervised variable selection, which displayed the largest CVIIM***s***,*n,K*-values in the real data analyses. The data-driven simulation design uses the ProstatecTranscr dataset and involves 2000 correlated normally distributed predictors. The methods and detailed results are presented in the Appendix A (Additional file 2).

Briefly, in the simulations the variance of CVIIM***s***,*n,K* as an estimator of CVIIM*P,n,K* was relatively high and decreased with decreasing CVIIM*P,n,K*-values. The bias was negligible. When displaying the CVIIM***s***,*n,K*-values graphically in the section Illustration we added error bars representing the variability of the (untruncated) CVIIM*P,n,K*-estimates from individual repetitions of CV. Our assumption that this variability measure also reflects the actual variance of CVIIM***s***,*n,K* was confirmed by the simulation, whereby this similarity in behavior was most pronounced for *K*=3. This indicates that the error bars obtained for the small *K*-values—of all considered values of *K* (see the section Further issues)—are the most appropriate for comparing the variability of individual CVIIM***s***,*n,K*-values.

### Further issues

In the section Outlook: other preparation steps we used a limited number of datasets in our analyses and noted that the results should thus not be over-interpreted. The results from the normalization and PCA analyses, in contrast, were based on 12 and 20 datasets respectively, and are thus more reliable. As a rule of thumb we recommend using at least 10 datasets for analyses for which the goal is the evaluation of the impact of CV incompleteness for a particular preparation step. However, the number of datasets to consider of course depends on the heterogeneity of the datasets. Quite generally, the variability of the relative performances of different classification methods over different datasets has been found to be large in previous literature [21, 22]. We can frequently make analogous observations with respect to the distribution of the CVIIM estimates over datasets. When studying these distributions, we can implicitly also observe variability inherent in individual CVIIM estimates. This variability is probably hard to estimate, given that the estimator involves a fraction of two CV estimates, the variance of which is very difficult to estimate [23].

In CV the training sets are necessarily smaller than the whole dataset and the CV error estimate is thus an upwardly biased estimator of the error of the prediction rule fit on the whole dataset. This type of bias also affects the relationship between E[*e*_*full*,*K*_(***S***)] and E[*e*_*incompl*,*K*_(***S***)]. Since in E[*e*_*incompl*,*K*_(***S***)] the considered analysis step(s) is/are performed on the whole dataset, the corresponding parameters are estimated more accurately than in E[*e*_*full*,*K*_(***S***)] due to the difference in sample sizes. This leads to a greater upward bias of *e*_*full*,*K*_(***s***) compared to *e*_*incompl*,*K*_(***s***) with respect to the prediction error of the prediction rule fit on the whole dataset. This can occasionally result in increased CVIIM***s***,*n,K* values. A strong decrease of the CVIIM estimates with increasing value of *K* is an indication of the presence of this problem. This is because for increasing *K* the size of the training sets gets closer to the full sample size, thereby diminishing the additional upward inherent bias of *e*_*full*,*K*_(***s***). In most of our analyses we observed no substantial dependence on *K*. We nevertheless recommend estimating CVIIM for several values of *K* as a form of sensitivity analysis, as done in our analyses.

For larger datasets the result of any preliminary step is expected to be more stable, and in fact results approach being deterministic as the sample size tends to infinity. Therefore with larger sample sizes the result of a preliminary step will be less affected when it is conducted on the whole dataset compared to the correct separation of training and test data. Thus CVIIM depends negatively on the sample size. In Figures S10, S11 and S12 in Appendix E (Additional file 2) for each investigated preparation step we plotted the dataset-specific means of the CVIIM-estimates over all respective settings against the sample sizes of the datasets. Here we clearly observe such a dependency: for large datasets (*n*∼100) the CVIIM-estimates were much smaller in most cases. This was also observed in the simulation study.

In practice, data preparation often consists of a combination of several preliminary steps, often with a natural ordering. For example, normalization of microarray data has to be performed before variable selection. There are, however, also cases with no predefined ordering. For example, dichotomization might be conducted before or after variable selection. Given a specific ordering of the steps, if one step is performed during CV, for obvious technical reasons one also has to perform all subsequent steps during CV. Of course it is also possible to compute CVIIM***s***,*n,K* globally for the whole combination of steps. In Appendix F (Additional file 2) we consider an example of such a combination. In this example a single analysis step was mainly responsible for the difference between *e*_*full*,*K*_(***s***) and *e*_*incompl*,*K*_(***s***).

CVIIM is in its current form only applicable to binary classification problems. It can however be easily adjusted to many other regression problems by replacing the misclassification errors in Eq. (1) by alternative error measures. The only requirement is that the loss function associated with the respective error type has positive range. Most common loss functions fulfill this requirement, for example the quadratic or absolute loss for linear regression, the integrated Brier score for survival data, the check function in the case of quantile regression or the negative log-likelihood as an alternative to the error rate when the response variable is discrete.

Note again that CV provides dataset-internal error estimation. Consequently it estimates the error expected on observations which follow the same distribution as the training data. When a different dataset is used for evaluating the prediction rule—as done in external validation—the error can be expected to be higher [24]. CV can be used in the process of obtaining an adequate prediction rule when no external data is available, but before ultimately applying a prediction rule in medical practice it must be externally validated [25, 26].

## Conclusions

In conclusion, the empirical study using our new measure of CV incompleteness suggests that 1) RMA normalization and RMA normalization in combination with global VSN can be safely performed as preliminary data preparation steps on the whole dataset, since they yielded very small CVIIM-values for all 12 analyzed real datasets; 2) PCA has to be performed anew in each CV iteration—i.e. re-trained on each training set—to protect against a potential optimistic bias, since it yielded large CVIIM values in some of the 20 analyzed real datasets. The latter result shows that non-supervised data preparation steps can also lead to over-optimistic error estimation if performed before CV. Given the ubiquitous use of RMA in microarray analysis it is reassuring that the common practice of performing RMA before CV is not harmful.

Due to the complexity of modern biological data, traditional model assessment tools are often not appropriate or even employable and CV is the method of choice in evaluating prediction models. It is thus especially important to have reliable guidelines for its application. Moreover, data preparation is becoming increasingly important, especially for data generated by high-throughput technologies. The need to empirically evaluate the impact of CV incompleteness with regard to these data preparation steps likewise increases. Our paper illustrates—through the application to important data preparation steps—that CVIIM is a useful tool in this endeavor.

## Additional files

Additional file 1
**Zipped folder containing all necessary R-Code to reproduce and evaluate the real-data analyses and simulations presented in the paper and in Additional file** 2 **, as well as Rda-files containing all fold errors computed in the real-data analysis enabling fast evaluation of the corresponding results.** (ZIP16691 kb)

Additional file 2
**Pdf-file containing all Supporting Information referenced in the paper.** A. Simulation study for the example of supervised variable selection. B. Methodological background. C. Normalization study: Dependency of CVIIM***s***,*n,K* on *e*_*full*,*K*_(***s***). D. Other preparation steps. E. Dependency of CVIIM on sample size. F. Combination of several steps. (PDF 276 kb)

## Abbreviations

CV: Cross-validation
CVIIM: CV incompleteness impact measure
INIMEs: iterationwise non-truncated incompleteness measure estimates
LDA: Linear discriminant analysis
NSC: Nearest shrunken centroids
PCA: Principal component analysis
PLS-LDA: LDA performed on partial least squares components
RF: Random forest
RMA: Robust multi-array average
RMAglobalVSN: RMA where the quantile-normalization step is expanded by VSN without calibration
VSN: Variance stabilization normalization

## References

[1] Simon R, Radmacher MD, Dobbin K, McShane LM (2003). Pitfalls in the use of dna microarray data for diagnostic and prognostic classification. J Nat Cancer Inst.

[2] Daumer M, Held U, Ickstadt K, Heinz M, Schach S, Ebers G (2008). Reducing the probability of false positive research findings by pre-publication validation—experience with a large multiple sclerosis database. BMC Med Res Methodol.

[3] Ambroise C, McLachlan GJ. Proc Nat Acad Sci USA. 2002; 99:6562–6. 10.1073/pnas.102102699 PMC124442 11983868

[4] Wood IA, Visscher PM, Mengersen KL (2007). Classification based upon gene expression data: bias and precision of error rates. Bioinformatics.

[5] Zhu JX, McLachlan GJ, Jones LB-T, Wood IA (2008). On selection biases with prediction rules formed from gene expression data. J Stat Plann Inference.

[6] Varma S, Simon R (2006). Bias in error estimation when using cross-validation for model selection. BMC Bioinformatics.

[7] Bernau C, Augustin T, Boulesteix AL (2013). Correcting the optimal resampling-based error rate by estimating the error rate of wrapper algorithms. Biometrics.

[8] Boulesteix AL, Strobl C (2009). Optimal classifier selection and negative bias in error rate estimation: an empirical study on high-dimensional prediction. BMC Med Res Methodol.

[9] Westerhuis JA, Hoefsloot HCJ, Smit S, Vis DJ, Smilde AK, van Velzen EJJ, van Duijnhoven JPM, van Dorsten FA (2008). Assessment of plsda cross validation. Metabolomics.

[10] Hastie T, Tibshirani R, Friedman J (2009). The Elements of statistical learning: data mining, inference and prediction.

[11] Zhu X, Ambroise C, McLachlan GJ (2006). Selection bias in working with the top genes in supervised classification of tissue samples. Stat Methodol.

[12] Irizarry RA, Hobbs B, Collin F, Beazer-Barclay YD, Antonellis KJ, Scherf U (2003). Exploration, normalization, and summaries of high density oligonucleotide array probe level data. Biostatistics.

[13] Kolesnikov N, Hastings E, Keays M, Melnichuk O, Tang YA, Williams E, et al. ArrayExpress update – simplifying data submissions. Nucleid Acid Res. 2015. doi:10.1093/nar/gku1057. 10.1093/nar/gku1057 PMC4383899 25361974

[14] Barrett T, Wilhite SE, Ledoux P, Evangelista C, Kim IF, Tomashevsky M (2013). Ncbi geo: archive for functional genomics data sets–update. Nucleid Acids Res.

[15] Singh D, Febbo PG, Ross K, Jackson DG, Manola J, Ladd C (2002). Gene expression correlates of clinical prostate cancer behavior. Cancer Cell.

[16] Bin RD, Herold T, Boulesteix AL (2014). Added predictive value of omics data: specific issues related to validation illustrated by two case studies. BMC Med Res Methodol.

[17] Kostka D, Spang R (2008). Microarray based diagnosis profits from better documentation of gene expression signatures. PLoS Comput Biol.

[18] Huber W, von Heydebreck A, Sültmann H, Poustka A, Vingron M (2002). Variance stabilization applied to microarray data calibration and to the quantification of differential expression. Bioinformatics.

[19] Huber W. Introduction to robust calibration and variance stabilisation with VSN. Vignette. 2014. http://www.bioconductor.org/packages/release/bioc/vignettes/vsn/inst/doc/vsn.pdf/. Accessed 13 Feb 2015.

[20] Dai JJ, Lieu L, Rocke D (2006). Dimension reduction for classification with gene expression microarray data. Stat Appl Genet Mol Biol.

[21] Boulesteix AL, Hable R, Lauer S, Eugster MJE (2015). A statistical framework for hypothesis testing in real data comparison studies. Am Stat.

[22] Boulesteix AL (2013). On representative and illustrative comparisons with real data in bioinformatics: response to the letter to the editor by Smith et al.. Bioinformatics.

[23] Bengio Y, Grandvalet Y (2004). No unbiased estimator of the variance of k-fold cross-validation. J Mach Learn Res.

[24] Bernau C, Riester M, Boulesteix AL, Parmigiani G, Huttenhower C, Waldron L (2014). Cross-study validation for the assessment of prediction algorithms. Bioinformatics.

[25] Simon R (2004). When is a genomic classifier ready for prime time?. Nat Clin Prac.

[26] Collins GS, de Groot JA, Dutton S, Omar O, Shanyinde M, Tajar A (2014). External validation of multivariable prediction models: a systematic review of methodological conduct and reporting. BMC Med Res Methodol.

