# Supplementary material for: A measure of the impact of CV incompleteness on prediction error estimation with application to PCA and normalization
Source: BMC Med Res Methodol. 2015 Nov 4;15:95. doi: 10.1186/s12874-015-0088-9 (PMC4634762; doi:10.1186/s12874-015-0088-9)
Supplement: Additional file 2 — Pdf-file containing all Supporting Information referenced in the paper. A. Simulation study for the example of supervised variable selection. B. Methodological background. C. Normalization study: Dependency of CVIIMs,n,K on e full,K(s). D. Other preparation steps. E. Dependency of CVIIM on sample size. F. Combination of several steps. (PDF 276 kb) [file 12874_2015_88_MOESM2_ESM.pdf]

Appendices to the
BMC Medical Research Methodology article:

A measure of the impact of CV incompleteness on prediction error estimation with application to PCA and normalization

Roman Hornung[*,1], Christoph Bernau[1,2], Caroline Truntzer[3], Rory Wilson[1], Thomas Stadler[4], and Anne-Laure Boulesteix[1]

[1] Department of Medical Informatics, Biometry and Epidemiology, University of Munich, Marchioninistr. 15, D-81377 Munich, Germany

[2] Leibniz Supercomputing Center, Boltzmannstr. 1, D-85748 Garching, Germany

[3] Clinical Innovation Proteomic Platform, Centre Hospitalo-Universitaire de Dijon, 15 Bd Maréchal de Lattre de Tassigny, F-21000 Dijon, France

[4] Department of Urology, University of Munich, Marchioninistr. 15, D-81377 Munich, Germany

[*]Correspondence: hornung@ibe.med.uni-muenchen.de

A Simulation study for the example of supervised variable selection

As described in the section 'Simulation study' of the main paper, we conducted a simulation study to investigate basic statistical properties of $\text{CVIIM}_{\boldsymbol{s},n,K}$.

Simulation setup

To make our simulation design representative of biomedical data, we use the transcriptomic dataset `ProstatecTranscr` to estimate realistic parameters to be used in the data-generating process. Datasets of sizes $n = 50$ and $n = 100$ are generated with $p = 2000$ continuous variables and a binary target variable with equal proportions in the classes.

We consider two different settings for the mean and covariance structure $MeanCov$ for the classes:

1. Scenario with strong signal:

   2000 correlated variables are generated, of which 200 are informative, these having class-specific means and covariances. As a first step towards obtaining the simulation setup we preselect 2000 of the 12625 variables of `ProstatecTranscr`, those yielding the smallest p-values from two-sample t-tests between the observations from the two classes. From these we again select the 200 variables corresponding to the smallest p-values: we take these as the informative variables, with the remaining 1800 as the non-informative. For each informative variable we calculate the difference between the mean of the observations belonging to class 2 and that of the observations belonging to class 1, resulting in the vector $\hat{\boldsymbol{\delta}}$ of length 200. Furthermore we calculate the empirical covariance matrix of the informative variables separately for classes 1 and 2: $\hat{\boldsymbol{\Sigma}}_{\boldsymbol{class1}}$ and $\hat{\boldsymbol{\Sigma}}_{\boldsymbol{class2}}$. In the simulation, the vector of the informative variables is drawn from $N(\mathbf{0}_{200}, \hat{\boldsymbol{\Sigma}}_{\boldsymbol{class1}})$ for observations from class 1 and from $N(\hat{\boldsymbol{\delta}}, \hat{\boldsymbol{\Sigma}}_{\boldsymbol{class2}})$ for observations from class 2. We could theoretically draw the values of all 1800 non-informative variables at once from a multivariate normal distribution with covariance matrix estimated from the data. However, this is computationally intractable. Therefore we split the 1800 non-informative variables into blocks of 200 variables, and for each of these blocks we calculate the empirical covariance matrix $\hat{\boldsymbol{\Sigma}}_{\boldsymbol{0j}}$ $j = 1, \ldots, 9$. In the simulation we draw a vector from $N(\mathbf{0}_{200}, \hat{\boldsymbol{\Sigma}}_{\boldsymbol{0j}})$ for $j = 1, \ldots, 9$ and subsequentially combine these vectors. Thus, the covariance matrix of the non-informative variables is block-diagonal with a block size of 200.

2. Scenario with weak signal:

   This scenario is conceptually equivalent to the previous with the following differences: only 100 variables are informative, the entries in the mean vector $\hat{\boldsymbol{\delta}}$ for class 2 from scenario 1—corresponding to the 100 informative variables—are multiplied by 0.7 and the block sizes for the non-informative variables are reduced from 200 to 100.

For the supervised variable selection we consider two different numbers $psel$ of selected variables: $psel = 10$ and $psel = 1000$. The variables yielding the smallest p-values from two-sample t-tests between the observations from the two classes are selected. Linear Discriminant Analysis

is used as a classification method with $psel = 10$ variables and Diagonal Linear Discriminant Analysis is used with $psel = 1000$. Again we consider the following commonly used splitting ratios between the sizes of the training and test sets: 2:1 (3-fold CV), 4:1 (5-fold CV) and 9:1 (10-fold CV). K will again denote the number of folds in the CV.

We perform the simulation for each possible combination of $MeanCov$, n, $psel$ and K, leading to 24 simulation settings in total. For each setting we simulate 2000 datasets and for each we calculate the estimate $\mathrm{CVIIM}_{\boldsymbol{s},n,K}$. As with our real-data analyses we repeat the full and incomplete CV 300 times for each simulated dataset.

For approximating the true measure $\mathrm{CVIIM}_{P,n,K}$ we approximate both $\mathrm{E}[e_{full,K}(\boldsymbol{S})]$ and $\mathrm{E}[e_{incompl,K}(\boldsymbol{S})]$ based on 10^5 simulated datasets of size n. Each dataset is randomly split into a training set of size $n_{train} := \lceil n(K-1)/K \rceil$ and a test set of size $n - n_{train}$. In the b-th iteration ($b = 1, \ldots, 10^5$) the approximation is done as follows. For $\mathrm{E}[e_{full,K}(\boldsymbol{S})]$ the variable selection and the training of the classifier are performed on the training set only and the resulting classifier is subsequently applied to the test set to calculate the error rate. For $\mathrm{E}[e_{incompl,K}(\boldsymbol{S})]$ we proceed in the same way except that the variable selection is performed on the whole dataset. By averaging over the 10^5 simulated datasets, we can expect to obtain close approximations of the true values of $\mathrm{E}[e_{full,K}(\boldsymbol{S})]$ and $\mathrm{E}[e_{incompl,K}(\boldsymbol{S})]$.

Results

Supplementary Figure 1 shows boxplots of the $\mathrm{CVIIM}_{\boldsymbol{s},n,K}$-values for all simulation settings. The bias with respect to the true measure values $\mathrm{CVIIM}_{P,n,K}$ is negligible in all settings. However, the variance around the true values is relatively large in many of the considered settings. Further note that when computing CVIIM over multiple datasets—as one would in a more extensive real-data analysis—the variability measured takes into account that within the given distribution (as examined in this simulation study) and that over the datasets.

The dependency of $\mathrm{CVIIM}_{P,n,K}$ on the individual simulation parameters can be better assessed by examining Supplementary Figure 2. The number of observations n has a negative effect on CVIIM in all cases. An important and slightly surprising observation is that our results suggest no or only a slight dependence on the number of folds K. We observe higher values of CVIIM when selecting 1000 variables, but this should not be over-interpreted since it may result from the specific simulation design. In our supervised variable selection analyses on real datasets we did not make this observation. The influence of the mean-covariance structure $MeanCov$ depends on $psel$ (see Supplementary Figure 1). For $psel = 10$ we observe smaller $\mathrm{CVIIM}_{\boldsymbol{s},n,K}$-values for the scenario with weak effects compared to that with strong effects; for $psel = 1000$ it is the reverse. This might be explained by the fact that in the scenario with weak effects there are only 100 informative variables. When selecting 1000 variables more noise variables are selected, impacting $\mathrm{E}[e_{full,K}(\boldsymbol{S})]$—causing the error to be larger—much more than $\mathrm{E}[e_{incompl,K}(\boldsymbol{S})]$.

The dependency of the variance of $\mathrm{CVIIM}_{\boldsymbol{s},n,K}$ on the simulation parameters and on $\mathrm{CVIIM}_{P,n,K}$ is visualized in Supplementary Figure 3. Unsurprisingly the variance decreases with increasing n and increases with the number of folds K. The latter can be explained as follows: $\mathrm{CVIIM}_{\boldsymbol{s},n,K}$ involves the fraction of two CV estimators which, with increasing K, become increasingly dataset dependent—due to the training sets sharing more observations with the entire

dataset—and therefore more variable. In the scenario with the stronger effects we generally observe larger variances. When selecting only $psel = 10$ variables the variances are also much higher than for $psel = 1000$.

For the scenario with $psel = 10$ we observe a strong dependency of the variance on the true value of the measure, with smaller measure values leading to smaller variances. This dependency cannot be seen as clearly in the case of $psel = 1000$: a possible explanation is that the measure values are in general higher in this setting, obscuring the dependence at the—relatively—smaller CVIIM values.

The left panel of Supplementary Figure 4 visually suggests a dependence of $\text{CVIIM}_{P,n,K}$ on the true error $\text{E}[e_{full,K}(\boldsymbol{S})]$, meaning that the use of the ratio of the errors in the calculation of CVIIM might not be sufficient to eliminate such an undesirable dependency. However this observed dependency may be explained by two observations regarding the simulation design. Firstly, $\text{CVIIM}_{P,n,K}$ as well as $\text{E}[e_{full,K}(\boldsymbol{S})]$ is larger for the smaller sample size $n = 50$, corresponding to the upper part of each ellipse. This negative dependency on n is natural—see the section 'Further issues' of the main paper. Secondly, the upper right ellipse—being responsible for much of the observed positive dependency of $\text{CVIIM}_{P,n,K}$ on $\text{E}[e_{full,K}(\boldsymbol{S})]$—contains all scenarios with both weak effects and $psel = 1000$. As stated above, we suppose that the higher number of noisy variables inherent to $psel = 1000$ is responsible for the increase in $\text{E}[e_{full,K}(\boldsymbol{S})]$ and $\text{CVIIM}_{P,n,K}$. The plot in the right panel of Supplementary Figure 4 suggests a much stronger dependence of $\max(\ \text{E}[e_{full,K}(\boldsymbol{S})] - \text{E}[e_{incompl,K}(\boldsymbol{S})],\ 0\ )$ on the true error. Here the values corresponding to the weak signal are also larger for $psel = 10$ and in the case of $psel = 1000$ the difference between the weak and strong signals is much bigger than for $\text{CVIIM}_{P,n,K}$.

In the section 'Illustration' in the main paper and in Appendix D we attempt to reflect the variance of $\text{CVIIM}_{\boldsymbol{s},n,K}$ through the use of the 25%- and 75%-quantiles of $\text{CVIIM}_{\boldsymbol{s},n,K,b} = 1 - e_{incompl,K}(\boldsymbol{s})_b / e_{full,K}(\boldsymbol{s})_b$, where the index b indicates that these errors were obtained for run b (with $b = 1, \dots, B$). Using the simulation results we can investigate whether the variability of the $\text{CVIIM}_{\boldsymbol{s},n,K,b}$-values is indeed a meaningful surrogate for the variance of $\text{CVIIM}_{\boldsymbol{s},n,K}$. As a measure for the variability of the $\text{CVIIM}_{\boldsymbol{s},n,K,b}$-values, for each simulated dataset we calculate the empirical variance of the $\text{CVIIM}_{\boldsymbol{s},n,K,b}$-values ($b = 1, \dots, 300$), defining it as the "observed variability". In Supplementary Figure 5 the values of the observed variability are plotted against the (approximated) true variance. Plots on the log-scale are also provided to enable comparisons of the small boxplots. In all plots we observe that the variance of the observed variability gets larger with a larger true variance. The results moreover clearly suggest that the size of the observed variability is also strongly and positively influenced by the size of the true variance. This dependency seems to be strongest for $K = 3$ and weakest for $K = 10$. This observed diminished relation between observed variability and actual variance with increasing value of K, becomes clearer when considering a fundamental shortcoming of the observed variability, which inhibits an even stronger relation to the actual variance. The observed variability does not account for the fact that the error estimates in the B (incomplete) CV repetitions are dependent. For smaller training set sizes the individual CV estimates in $e_{full,K}(\boldsymbol{s})$ as well as in $e_{incompl,K}(\boldsymbol{s})$ are less similar, i.e., less dependent. In these cases the observed variability thus better reflects the true variance. In contrast, in the case of larger training set sizes, the stronger dependency makes the behavior of the actual variance more different from that of the observed variability. These results

suggest that the error bars obtained for the small K-values are most appropriate for comparing the variability of individual $\mathrm{CVIIM}_{\boldsymbol{s},n,K}$-values.

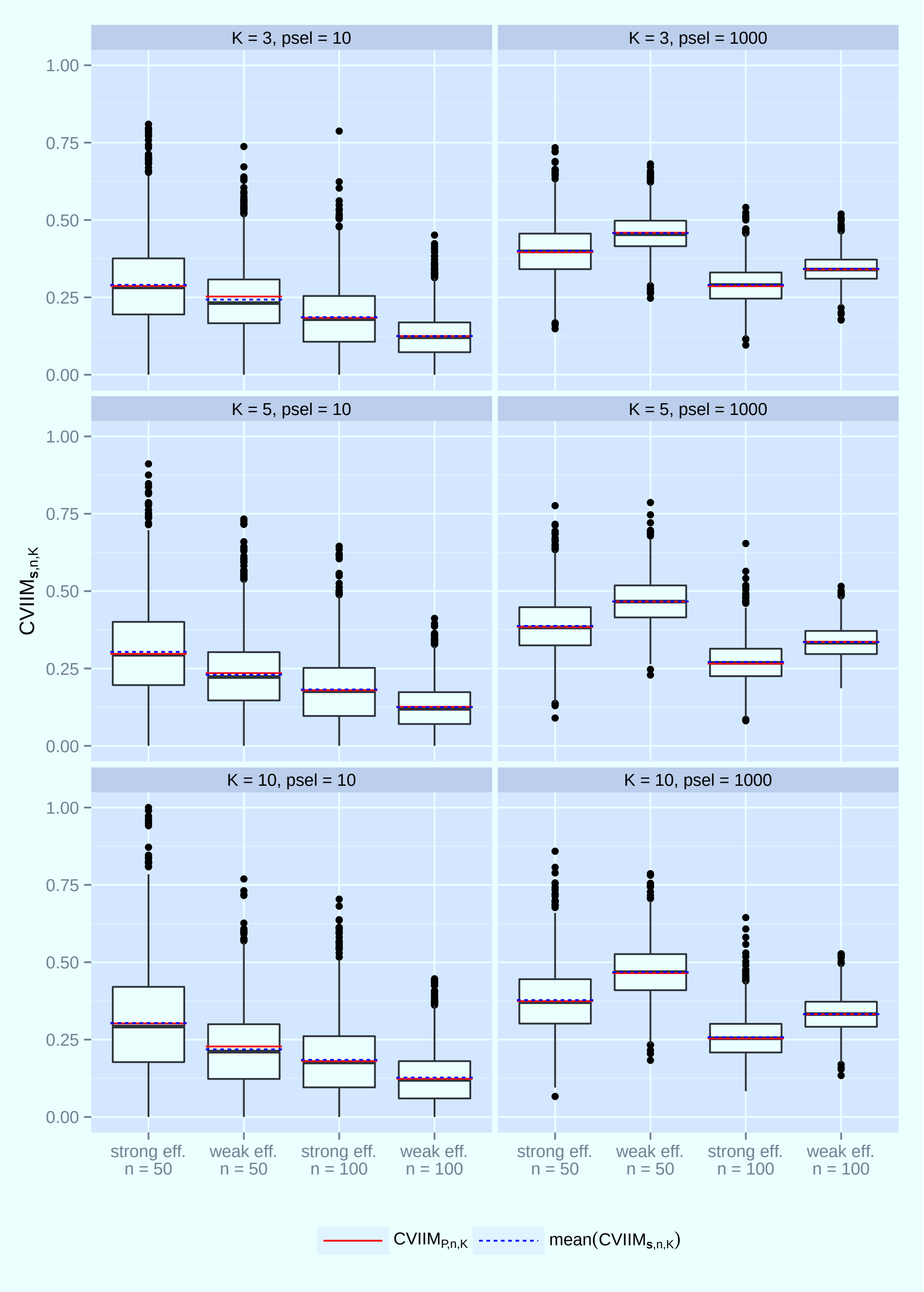

Supplementary Figure 1: Estimates $\mathrm{CVIIM}_{\boldsymbol{s},n,K}$ from all simulation iterations and true $\mathrm{CVIIM}_{P,n,K}$-values (solid lines)

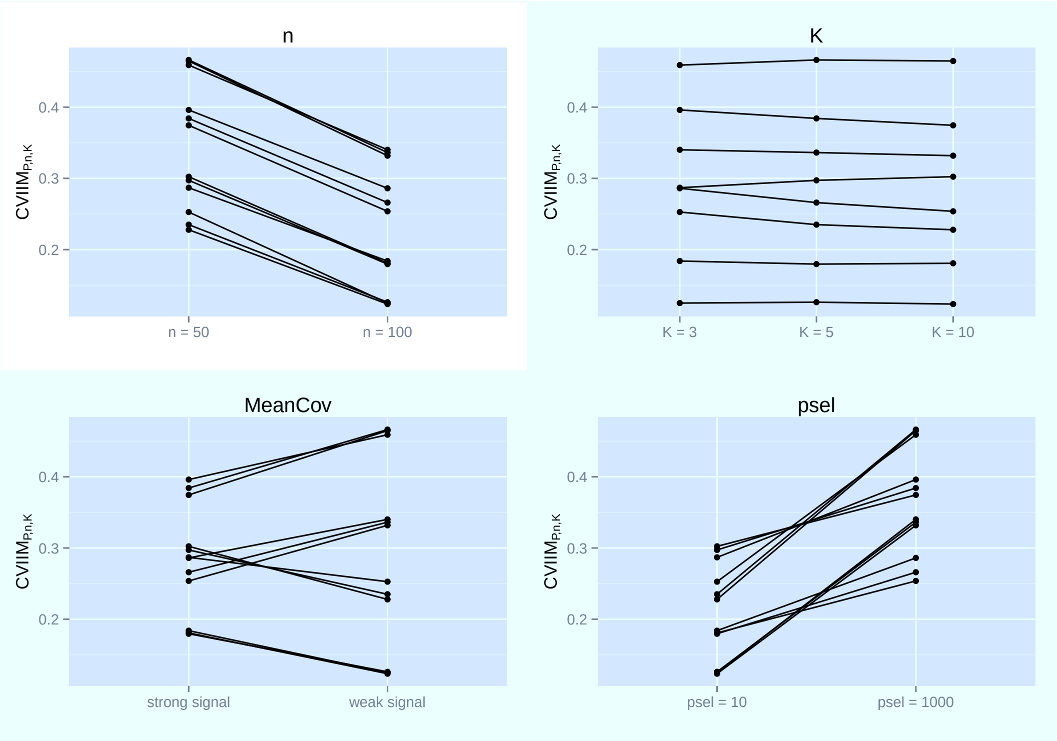

Supplementary Figure 2: $\text{CVIIM}_{P,n,K}$-values for different parameter settings, grouped according to n (top left), K (top right), $MeanCov$ (bottom left), and $psel$ (bottom right)

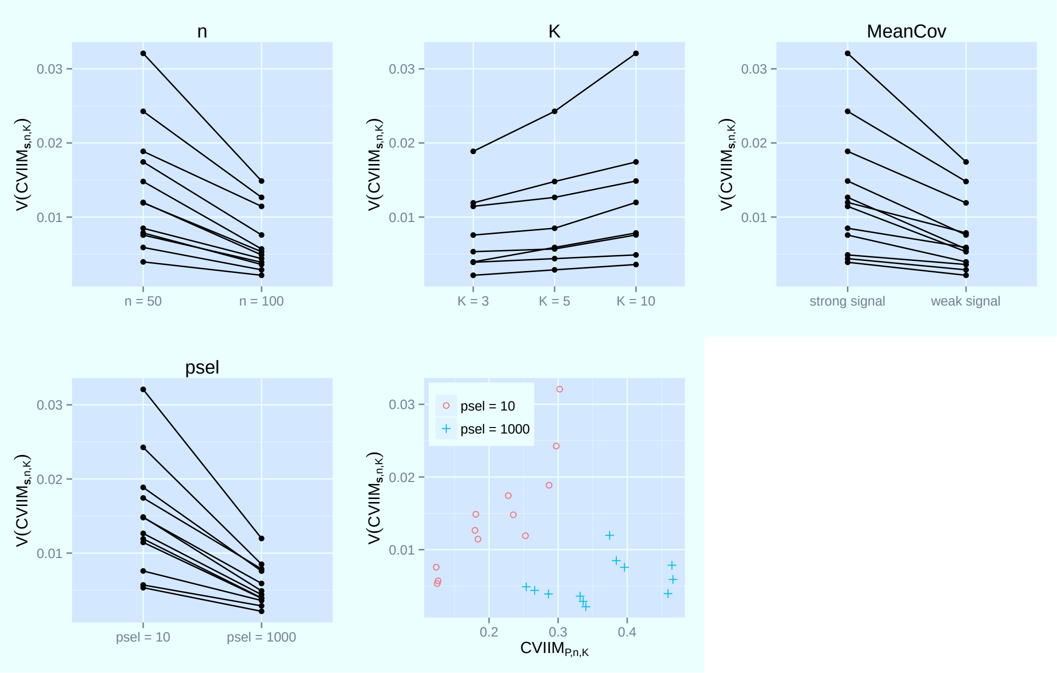

Supplementary Figure 3: (Approximated) true variances of $\text{CVIIM}_{\boldsymbol{s},n,K}$ for different parameter settings, grouped according to n, K, $MeanCov$ and $psel$, and scatterplot of the variance of $\text{CVIIM}_{\boldsymbol{s},n,K}$ vs. the true $\text{CVIIM}_{P,n,K}$-values. The true variances are approximated by the empirical variances over the 2000 simulation iterations

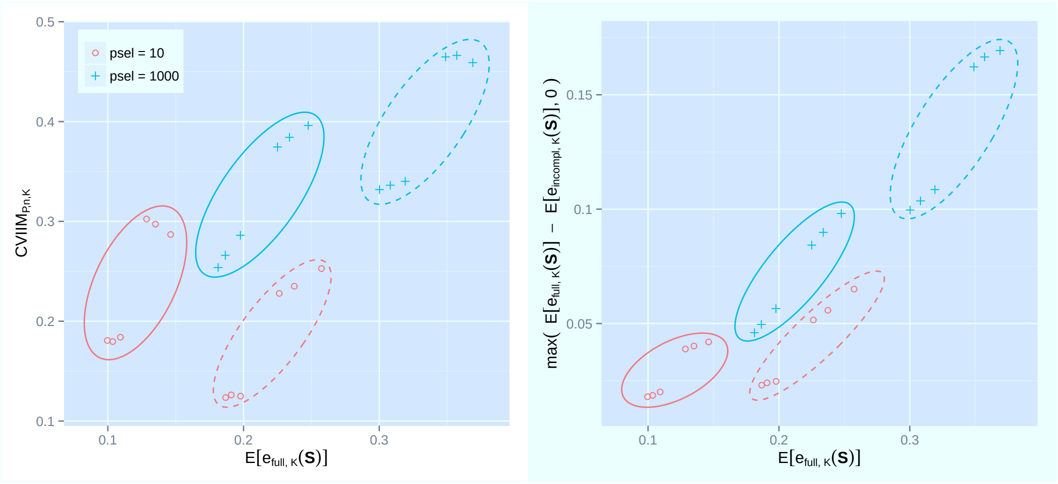

Supplementary Figure 4: Values of $\text{CVIIM}_{P,n,K}$ (left) and $\text{E}[e_{full,K}(\boldsymbol{S})] - \text{E}[e_{incompl,K}(\boldsymbol{S})]$ (right) plotted against true errors. Each solid ellipse contains values corresponding to the strong signal and each dashed ellipse values corresponding to the weak signal

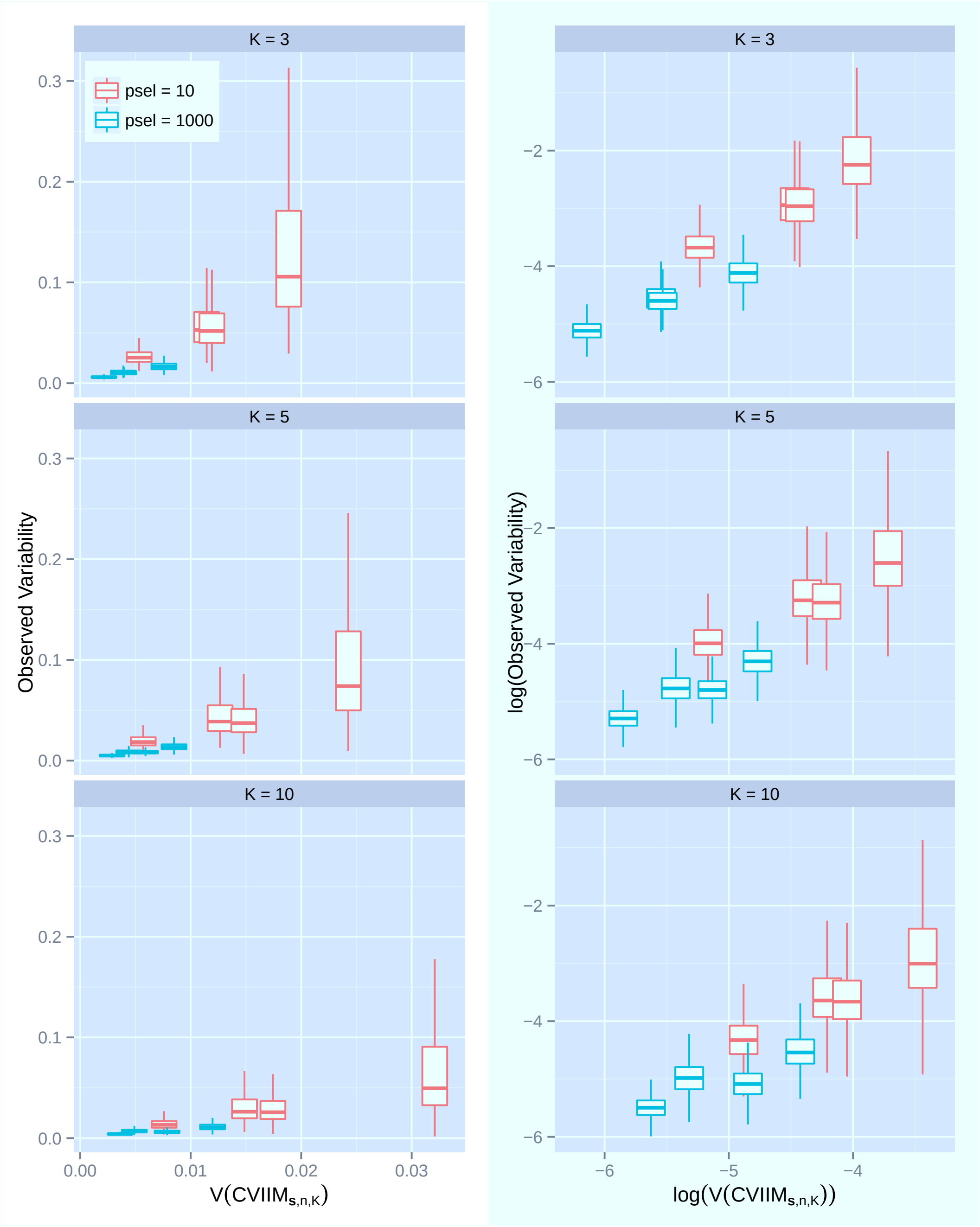

Supplementary Figure 5: (Log-) values of the "observed variability" plotted against the actual (log-) variance of $\text{CVIIM}_{\boldsymbol{s},n,K}$ for different values of K

B Methodological background

B.1 Prediction rules, prediction errors and their estimation

Let $\mathcal{X} \subset \mathbb{R}^p$ denote the predictor space and $\mathcal{Y} = \{1, 2\}$ the space of the response variable. Note that this notation also allows for categorical covariates, e.g. $\mathcal{X} = \{0, 1\}^p \subset \mathbb{R}^p$ in the case of dummy-coded categorical predictors. Let $\boldsymbol{S} = \{(\boldsymbol{X}_1, Y_1), \ldots, (\boldsymbol{X}_n, Y_n)\}$ be an *i.i.d.* random sample with observations drawn from the distribution P. Most importantly, in our paper $\boldsymbol{x} \in \mathcal{X}$ denotes the "raw" data, meaning that these predictors may be subject to data preparation steps (possibly modifying their number or scale of measurement) before being used as predictors in classification.

We consider a classification function $g : \mathcal{X} \mapsto \mathcal{Y}$, $\boldsymbol{x} \mapsto g(\boldsymbol{x})$ that takes the vector $\boldsymbol{x}$ as an argument and returns a prediction y of the value of the response variable. For example, consider a classification task where, based on microarray samples, patients are classified as having cancer or not using the Nearest Shrunken Centroids approach [1]. Then the corresponding function g would take the pre-normalized expression values as an argument, perform normalization and classify the sample using a certain value of the shrinkage parameter. These steps are assumed to be performed in an ideal way, where all occurring parameters are estimated or optimized using a hypothetical dataset with sample size tending to infinity.

In practice g is estimated from the available data. We therefore define $\hat{g}_{\boldsymbol{S}} : \mathcal{X} \mapsto \mathcal{Y}$, $\boldsymbol{x} \mapsto \hat{g}_{\boldsymbol{S}}(\boldsymbol{x})$ as the classification function estimated from $\boldsymbol{S}$. In the example outlined above, this means that the parameters involved in the normalization procedure, as well as the averages and variances involved in the Nearest Shrunken Centroids classifier, are estimated from $\boldsymbol{S}$ and that the shrinkage parameter is also chosen based on $\boldsymbol{S}$. The estimated classification function $\hat{g}_{\boldsymbol{S}}$ can then be used to predict y for a new observation.

Note that—as already outlined in the section 'Addon procedures' of the main paper—depending on the procedures involved in the estimation, it is not always straightforward to construct such a function $\hat{g}_{\boldsymbol{S}}$ that can be applied to predict independent data. From now on, we will however assume that the necessary addon procedures (see the section 'Addon procedures' of the main paper) are available and that we can thus construct the function $\hat{g}_{\boldsymbol{S}}$.

It is important to assess the prediction error of $\hat{g}_{\boldsymbol{S}}$, which is defined as

$$\varepsilon[\hat{g}_{\boldsymbol{S}}] := \mathrm{E}_{(\boldsymbol{X},Y)\sim P}[L(\hat{g}_{\boldsymbol{S}}(\boldsymbol{X}), Y)] = \int_{\mathcal{X}\times\mathcal{Y}} L(\hat{g}_{\boldsymbol{S}}(\boldsymbol{x}), y)\, dP(\boldsymbol{x}, y), \tag{1}$$

where $L(\cdot, \cdot)$ is an appropriate loss function, for example the indicator loss yielding the misclassification error rate, as used in the main paper. The error defined in Eq. (1) is commonly termed "conditional" because it refers to the specific sample $\boldsymbol{S}$. The *average error* over all samples following P^n is referred to as the unconditional error and denoted by $\varepsilon(n) := \mathrm{E}_{\boldsymbol{S}\sim P^n}[\,\varepsilon[\hat{g}_{\boldsymbol{S}}]\,]$.

Let $\boldsymbol{s} = \{(\boldsymbol{x}_1, y_1), \ldots, (\boldsymbol{x}_n, y_n)\}$ denote a realization of the random sample $\boldsymbol{S}$. If we had a large independent sample at hand we could estimate $\varepsilon[\hat{g}_{\boldsymbol{s}}]$ directly by comparing the true values of the response variable in this data to the predictions made by $\hat{g}_{\boldsymbol{s}}$. Having only $\boldsymbol{s}$ at hand a naive approach would be to estimate $\varepsilon[\hat{g}_{\boldsymbol{s}}]$ using $\boldsymbol{s}$ itself as test data. This approach yields the so-called "apparent error" or "resubstitution error" that is well known to be downwardly biased (i.e., too optimistic) as an estimator of $\varepsilon[\hat{g}_{\boldsymbol{s}}]$, since estimation of the classification function and error estimation are conducted on the same data. Resampling-based error estimation can be performed

to address this issue. The sample $\boldsymbol{s}$ is iteratively split into non-overlapping training and test datasets. In each iteration the function g is estimated based on the training set, and the error of this estimated function is assessed based on the test set. We examine K-fold cross-validation—the most widely used of these resampling-based approaches.

Given a random partition of the dataset $\boldsymbol{s}$ into K approximately equally sized folds $\boldsymbol{s}_1, \ldots, \boldsymbol{s}_K$, the K-fold cross-validation error estimate is given as

$$\frac{1}{K}\sum_{k=1}^{K}\frac{1}{\#\boldsymbol{s}_k}\sum_{j\,\in\,\{i\,:\,(\boldsymbol{x}_i,y_i)\,\in\,\boldsymbol{s}_k\}} L(\hat{g}_{\boldsymbol{s}\setminus\boldsymbol{s}_k}(\boldsymbol{x}_j), y_j),$$

whereby $\#$ represents the cardinality, $\boldsymbol{s}\setminus\boldsymbol{s}_k$ is the training set in iteration k and $\boldsymbol{s}_k$ is the test set. Since this estimate (highly) depends on the considered random partition of the sample $\boldsymbol{s}$ into K folds, it is recommended to repeat this procedure $B > 1$ times and average the error estimates over the B repetitions. With $\boldsymbol{s}_{b1}, \ldots, \boldsymbol{s}_{bK}$ denoting the folds considered in the b-th repetition, the repeated K-fold cross-validation error estimate is given as

$$e_K(\boldsymbol{s}) = \frac{1}{B}\sum_{b=1}^{B}\frac{1}{K}\sum_{k=1}^{K}\frac{1}{\#\boldsymbol{s}_{bk}}\sum_{j\,\in\,\{i\,:\,(\boldsymbol{x}_i,y_i)\,\in\,\boldsymbol{s}_{bk}\}} L(\hat{g}_{\boldsymbol{s}\setminus\boldsymbol{s}_{bk}}(\boldsymbol{x}_j), y_j). \tag{2}$$

If, for simplicity, we assume that the $\boldsymbol{s}_{b1}, \ldots, \boldsymbol{s}_{bK}$ ($b = 1, \ldots, B$) are equally sized with size $n_{train,K} := \#\boldsymbol{s}\setminus\boldsymbol{s}_{bk}$ for $b \in \{1, \ldots, B\}$ and $k \in \{1, \ldots, K\}$, it can be easily seen that $e_K(\boldsymbol{s})$ is an unbiased estimator of $\varepsilon(n_{train,K})$ and therefore an upwardly biased estimator of $\varepsilon(n)$. This bias is called the "inherent bias" of CV in [2]. Note that the notation $e_K(\boldsymbol{s})$ does not reflect the fact that the repeated K-fold cross-validation error estimate depends on the random partitions in the B iterations. For our purposes we assume B to be chosen large enough so that this dependency can be ignored.

B.2 Incomplete versus full cross-validation

With the issue of incomplete cross-validation in mind, we introduce the notation

$$\hat{g}^{\boldsymbol{a}_2}_{\boldsymbol{a}_1} : \mathcal{X} \;\mapsto\; \mathcal{Y} \qquad \boldsymbol{x} \;\mapsto\; \hat{g}^{\boldsymbol{a}_2}_{\boldsymbol{a}_1}(\boldsymbol{x}) \qquad \boldsymbol{a}_1 \subseteq \boldsymbol{a}_2 \subseteq \boldsymbol{s}$$

to denote an estimated classification function that is estimated partly based on a sample $\boldsymbol{a}_2$ and partly based on a possibly smaller subsample $\boldsymbol{a}_1$ (i.e., one or several steps may be performed on a bigger sample). Returning to the example of microarray-based classification, it is common practice to run the normalization procedure—and often also the parameter tuning—based on the whole dataset $\boldsymbol{s}$, but to perform the training of the classifier within cross-validation, i.e., based only on the training set $\boldsymbol{s}\setminus\boldsymbol{s}_{bk}$ in each iteration k of each repetition b. In this scenario $\boldsymbol{a}_2$ would be the whole dataset $\boldsymbol{s}$ and in each CV iteration $\boldsymbol{a}_1$ would be the training set $\boldsymbol{s}\setminus\boldsymbol{s}_{bk}$.

With $\boldsymbol{a}_1 = \boldsymbol{s}\setminus\boldsymbol{s}_{bk}$ and $\boldsymbol{a}_2 = \boldsymbol{s}$ for $b = 1, \ldots, B$ and $k = 1, \ldots, K$, we obtain the incomplete CV

error estimate, which is downwardly biased as an estimator of $\varepsilon(n_{train,K})$:

$$e_{incompl,K}(\boldsymbol{s}) := \frac{1}{B}\sum_{b=1}^{B}\frac{1}{K}\sum_{k=1}^{K}\frac{1}{\#\boldsymbol{s}_{bk}}\sum_{j\,\in\,\{i\,:\,(\boldsymbol{x}_i,y_i)\,\in\,\boldsymbol{s}_{bk}\}} L(\hat{g}^{\boldsymbol{s}}_{\boldsymbol{s}\setminus\boldsymbol{s}_{bk}}(\boldsymbol{x}_j), y_j),$$

where the index "*incompl*" indicates that the whole sample $\boldsymbol{s}$ is used for at least part of the data analysis steps required for the estimation of g, and that the resulting CV procedure is thus incomplete. The estimator $e_{incompl,K}(\boldsymbol{s})$ is unbiased as an estimator of the *average incomplete error* $\varepsilon_{incompl}(n_{train,K}; n) := \mathrm{E}_{\boldsymbol{S}\sim P^n}[L(\hat{g}^{\boldsymbol{S}}_{\boldsymbol{S}_{train,K}}(\boldsymbol{X}_{n_{train,K}+1}), Y_{n_{train,K}+1})]$, with $\boldsymbol{S}_{train,K} = \{(\boldsymbol{X}_1, Y_1), \ldots, (\boldsymbol{X}_{n_{train,K}}, Y_{n_{train,K}})\}$ and $(\boldsymbol{X}_{n_{train,K}+1}, Y_{n_{train,K}+1})$ playing the role of an arbitrary test set observation. We assume exchangeability of the random observations in $\boldsymbol{S}$.

Furthermore, since by definition $\hat{g}^{\boldsymbol{s}\setminus\boldsymbol{s}_{bk}}_{\boldsymbol{s}\setminus\boldsymbol{s}_{bk}} = \hat{g}_{\boldsymbol{s}\setminus\boldsymbol{s}_{bk}}$, we obtain the usual repeated K-fold error estimate from Eq. (2) if we set $\boldsymbol{a}_1 = \boldsymbol{a}_2 = \boldsymbol{s}\setminus\boldsymbol{s}_{bk}$ for $k = 1, \ldots, K$, and $b = 1, \ldots, B$. This estimator is denoted by $e_{full,K}(\boldsymbol{s})$:

$$e_{full,K}(\boldsymbol{s}) := e_K(\boldsymbol{s}) = \frac{1}{B}\sum_{b=1}^{B}\frac{1}{K}\sum_{k=1}^{K}\frac{1}{\#\boldsymbol{s}_{bk}}\sum_{j\,\in\,\{i\,:\,(\boldsymbol{x}_i,y_i)\,\in\,\boldsymbol{s}_{bk}\}} L(\hat{g}^{\boldsymbol{s}\setminus\boldsymbol{s}_{bk}}_{\boldsymbol{s}\setminus\boldsymbol{s}_{bk}}(\boldsymbol{x}_j), y_j),$$

where the index "*full*" underlines that *all* steps of prediction rule construction are conducted within the CV procedure, i.e., using the training sets only.

For easier interpretation, in the main paper and in other sections of Additional file 2 we write $\mathrm{E}[e_{full,K}(\boldsymbol{S})]$ for $\varepsilon(n_{train,K})$ and $\mathrm{E}[e_{incompl,K}(\boldsymbol{S})]$ for $\varepsilon_{incompl}(n_{train,K}; n)$.

B.3 Behavior of $\mathrm{CVIIM}_{\boldsymbol{s},n,K}$ for small $\varepsilon_{full}(n_{train,K})$-values

For very small values of $\varepsilon_{full}(n_{train,K})$, extreme CVIIM estimates can occur (either zero or very high values). For very small values of $e_{full,K}(\boldsymbol{s})$, the CVIIM estimate is highly sensitive to relatively small differences between $e_{incompl,K}(\boldsymbol{s})$ and $e_{full,K}(\boldsymbol{s})$, which may be due at least partly to random fluctuations. For example, suppose that $e_{full,K}(\boldsymbol{s}) = 0.01$ and $e_{incompl,K}(\boldsymbol{s}) = 0.001$, then we would have $\mathrm{CVIIM}_{\boldsymbol{s},n,K} = 0.9$. Note however that such extremely large results are expected to be rare due to a mechanism related to regression toward the mean: considering the high variance of CV estimates, in many cases very small values of $e_{full,K}(\boldsymbol{s})$ are an underestimation of $\varepsilon_{full}(n_{train,K})$. In this case it is unlikely that $\varepsilon_{incompl}(n_{train,K}; n)$ is considerably more affected by underestimation. Thus in such a situation it is unlikely that $e_{incompl,K}(\boldsymbol{s})$ is much smaller than $e_{full,K}(\boldsymbol{s})$. Instead, the incomplete CV error estimator $e_{incompl,K}(\boldsymbol{s})$ is more likely to be closer to its mean than $e_{full,K}(\boldsymbol{s})$, thereby preventing an overly large CVIIM estimate.

C Normalization study: Dependency of $\mathrm{CVIIM}_{\boldsymbol{s},n,K}$ on $e_{full,K}(\boldsymbol{s})$

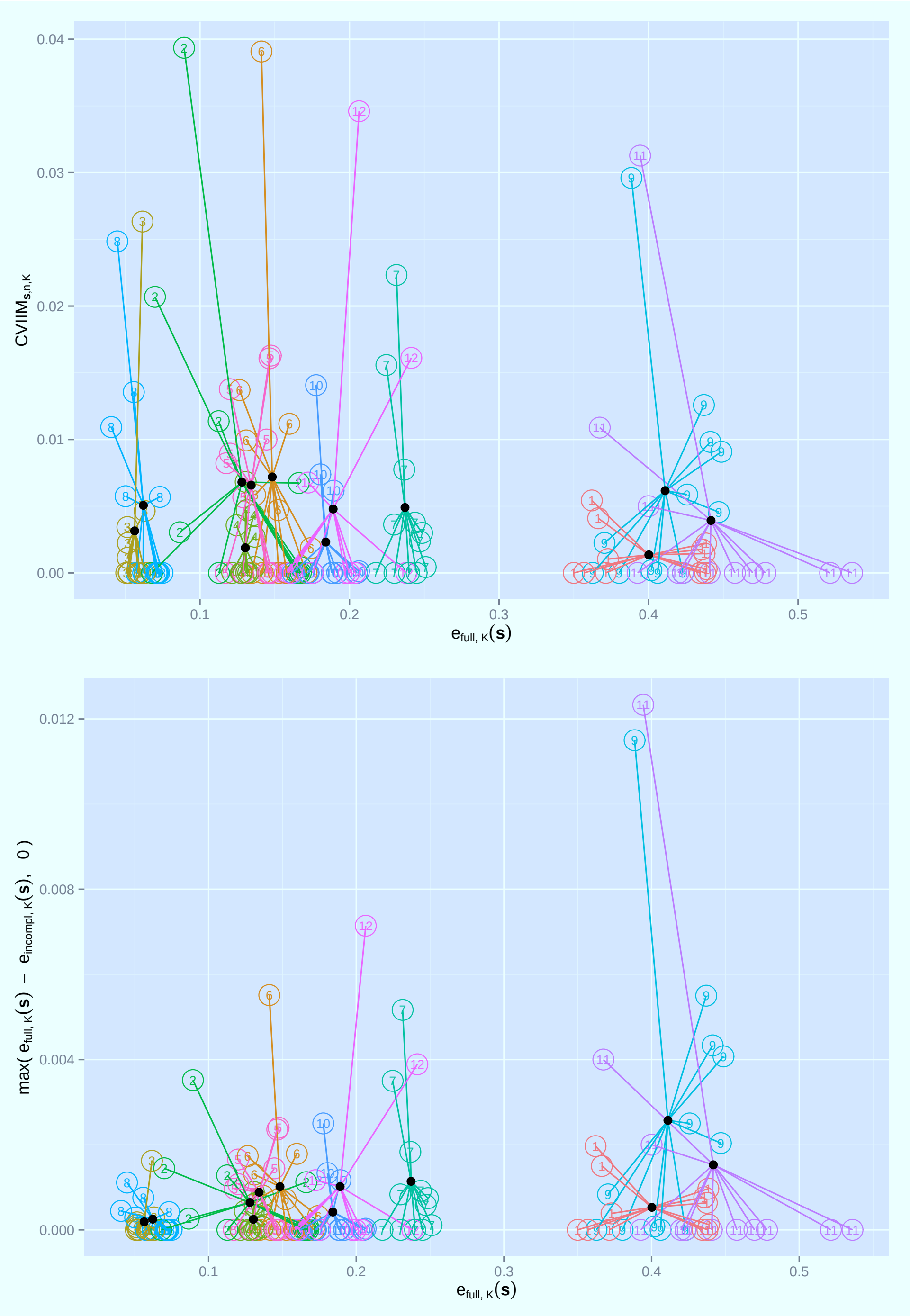

Supplementary Figure 6: Dependency on CV errors in normalization study. Upper panel: $\mathrm{CVIIM}_{\boldsymbol{s},n,K}$-values versus $e_{full,K}(\boldsymbol{s})$-values for all settings; Lower panel: Zero-truncated differences of $e_{full,K}(\boldsymbol{s})$- and $e_{incompl,K}(\boldsymbol{s})$-values versus $e_{full,K}(\boldsymbol{s})$-values for all settings. The colors and numbers distinguish the datasets. The filled black circles depict the respective means over the results of all settings obtained on the specific datasets.

D Other preparation steps

Optimization of tuning parameters An important data preparation step is the choice of tuning parameters. To illustrate the use of CVIIM in this context, we consider seven classification methods successively, each with one tuning parameter of interest optimized from a grid through internal CV as described in the section 'Study design' of the main paper: the number of iterations m_{stop} in componentwise boosting with logistic loss function (grid: $\{50, 100, 200, 500, 1000\}$) [3], the number of neighbors in the k-Nearest-Neighbors algorithm (kNN) (grid: $\{1, 2, \ldots, 10\}$), the L_1 shrinkage intensity in Lasso regression expressed as the fraction of the coefficient L_1-norm compared to the maximum possible L_1-norm (grid: $\{0.1, 0.2, \ldots, 0.9\}$) [4], the shrinkage intensity for the class centroids in Nearest Shrunken Centroids (grid: $\{0.1, 0.25, 0.5, 1, 2, 5\}$), the number of components in Linear Discriminant Analysis on Partial Least Squares components (grid: $\{1, 2, \ldots, 10\}$) [5], the number *mtry* of variables randomly sampled as candidates at each split in Random Forests (grid: $\{1, 5, 10, 50, 100, 500\}$) [6] and cost of constraints violation in Support Vector Machines with linear kernel (SVMs) (grid: $\{10^{-5} \cdot 40^{k/7} : k = 0, \ldots, 7\}$) [7].

We use the same four example datasets as in the illustrative application of CVIIM to supervised variable selection presented in the section 'Illustration' of the main paper. Supplementary Figure 7 and Supplementary Table 1 show the results. None of the methods exhibit large $\text{CVIIM}_{\boldsymbol{s},n,K}$-values. According to our rule of thumb only one result is classified as a medium effect—tuning of the number of components in PLS-LDA for dataset `GSE33205`—and the rest are classified as weak effects.

Researchers applying CV to optimize tuning parameters in the way described here may be tempted to use as an error estimate of the prediction rule that CV error estimate obtained during optimization for the ultimately chosen tuning parameter value, i.e. the smallest one. The optimistic bias of this estimate has been studied in the literature [2, 8, 9]. Given a large enough number of repetitions of the CV used in the optimization, this optimistic bias becomes equivalent to the bias resulting from performing the optimization before CV, as studied here. This is because the dependence of the CV error estimates—those of the optimization process—on the specific training/test set divisions diminishes with increasing number of repetitions. As a result, the additional distortion of the estimate studied by Varma and Simon [2] and Bernau et al. [8], which is due to the impact of optimally selecting the smallest error estimate, compared to the distortion of the incomplete CV estimate studied here, decreases in the same way.

We made an effort to choose reasonable parameter grids; however the above results are likely grid-dependent. Moreover, for methods such as Random Forests involving several important tuning parameters, it would be interesting to investigate the bias induced by incomplete CV when optimizing two or more tuning parameters simultaneously.

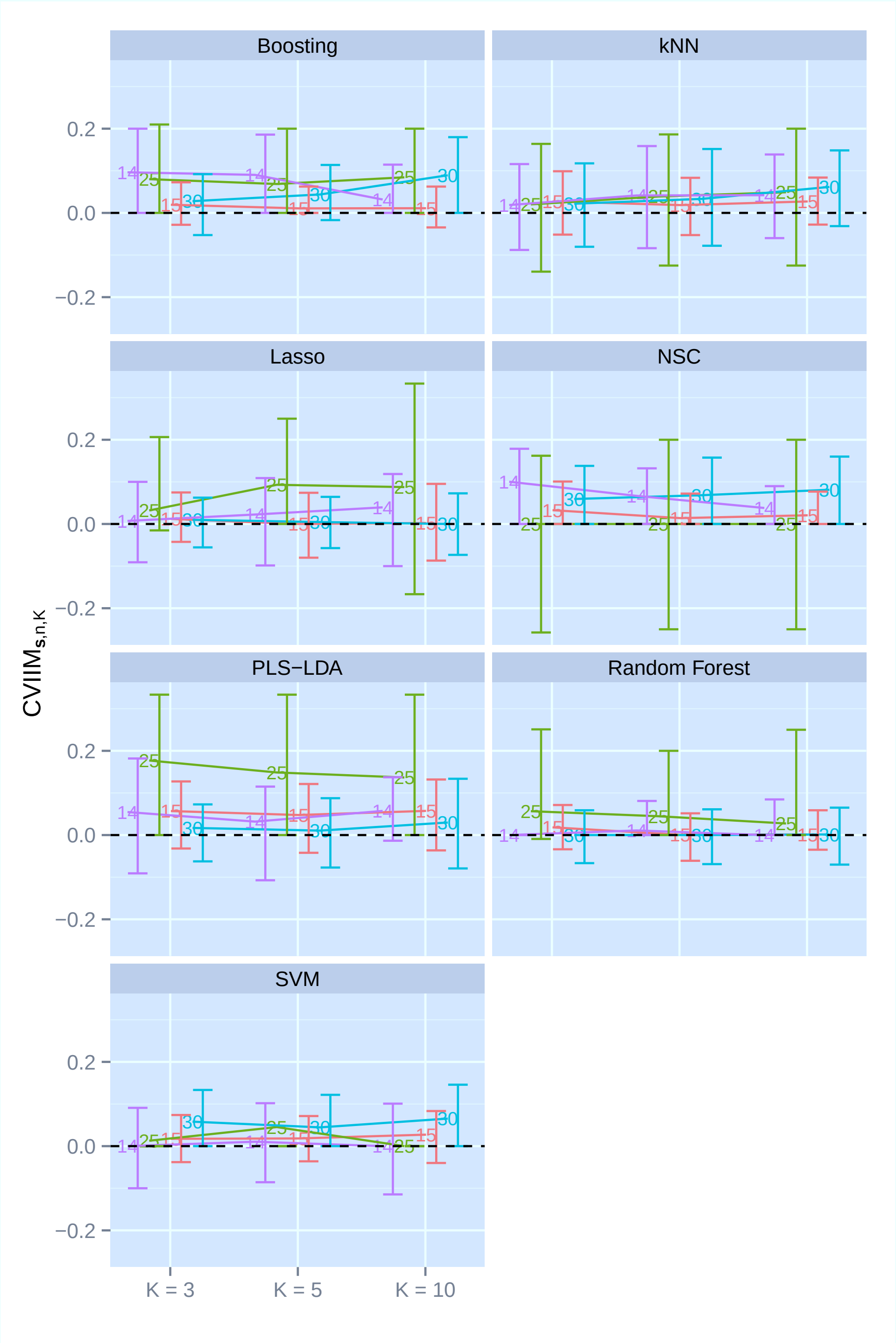

Supplementary Figure 7: $\mathrm{CVIIM}_{\boldsymbol{s},n,K}$-values from tuning study. The numbers distinguish the datasets.

Supplementary Table 1: Estimates of global CVIIM from the tuning study

number of sel. variables	$K = 3$	$K = 5$	$K = 10$
Boosting	0.0385	0.0415	0.0591
kNN	0.0227	0.0309	0.0465
Lasso	0.0124	0.0134	0.0126
NSC	0.0554	0.0472	0.0501
PLS-LDA	0.0456	0.0343	0.0486
Random Forest	0.0097	0.0014	0.0025
SVM	0.0350	0.0330	0.0417

Variable filtering by variance For each variable the empirical variance is calculated and half of the variables with the largest variances are selected. Such procedures are common in the context of gene expression data analysis. Their aim is to eliminate genes that exhibit little variation in their profile and are therefore generally not of interest [10]. We again use Diagonal Linear Discriminant Analysis as a classification method and the same four example datasets as in the case of supervised variable selection and tuning. Here we only observe zero or almost zero values. The global CVIIM estimates are correspondingly zero or (for $K = 3$) almost zero. These preliminary results suggest that the selection of a large number of variables in an unsupervised fashion might be performed outside CV, in contrast to supervised variable selection.

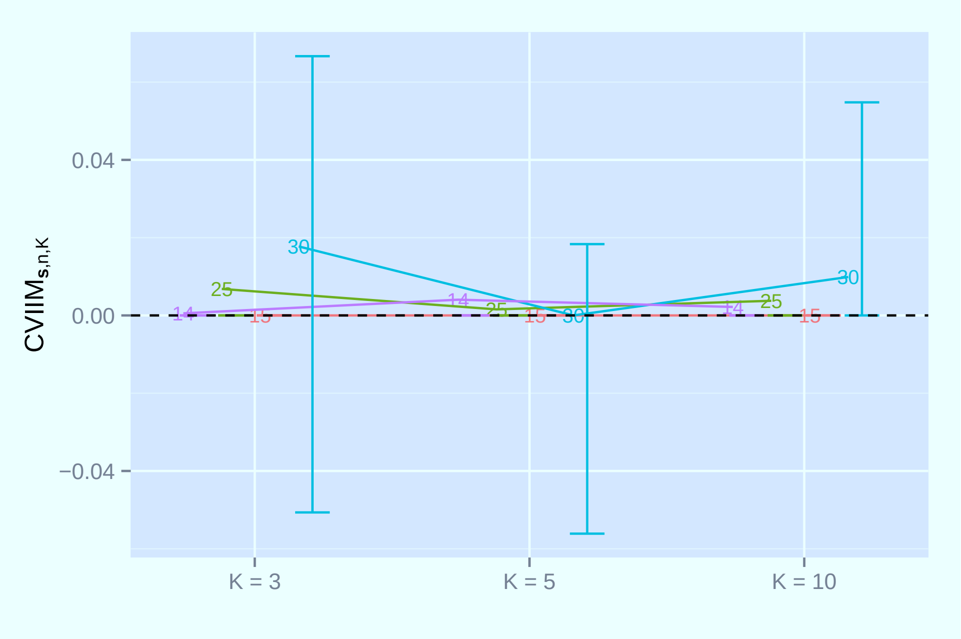

Supplementary Figure 8: $\text{CVIIM}_{\boldsymbol{s},n,K}$-values from variable filtering study. The numbers distinguish the datasets.

Imputation of missing values We perform k-Nearest-Neighbors imputation [11], a procedure that is commonly used for the analysis of high-dimensional microarray data. Prior to imputation

the variables are centered and scaled and the estimated means and standard deviations are stored. After imputing the values, they are rescaled using the stored standard deviations and the stored means are added to retransform the data to the original level. The result of the imputation may depend critically on the number k of nearest neighbors considered. Therefore to optimize this parameter on the grid $\{1, 2, 3, 5, 10, 15\}$ we again employ 3-fold CV. For correct addon imputation, in addition to using the means and standard deviations estimated from the training data, we also only examine the training data when searching for the k nearest neighbors.

For this illustrative study, we first consider a collection of five datasets, `GenitInfCow`, containing measurements on 51 cows, where 36 suffer from a major genital infection. Each dataset (IDs 33 - 37 in Table 1 in the main paper) contains measurements from a specific week and comprises between 21 and 27 variables. These datasets were obtained via personal communication with Michael Schmaußer. Random Forests are used as a classification method here. Our second example is the dataset `ProstatecMethyl` (ID 38 in Table 1), containing 222 variables obtained through DNA methylation profiling of 70 patients, of which 29 suffer from metastatic prostate cancer. This dataset was provided by the fifth author. Nearest Shrunken Centroids (NSC) are used as a classification method for this dataset. The shrinkage intensity (for NSC) and *mtry* (for Random Forests) are chosen by 3-fold CV.

The results, shown in Supplementary Figure 9, suggest that in this setting it seems to be irrelevant whether the considered imputation procedure is trained on the whole dataset or based on the training datasets only. The `GenitInfCow` datasets contain proportions of missing values between $\sim 8\%$ and $\sim 19\%$ with tendentiously lower proportions for more advanced weeks. This pattern is also reflected by the $\text{CVIIM}_{\boldsymbol{s},n,K}$-values, where we observe decreasing values for more advanced weeks, with the highest values being observed for the Dataset 33, that with the greatest number of missing values. The high-dimensional dataset `ProstatecMethyl` yields $\text{CVIIM}_{\boldsymbol{s},n,K}$-values of zero for all K-values. In this dataset only $\sim 3\%$ of values were missing, which is—although small compared to the `GenitInfCow` datasets—a proportion within the range of proportions likely to occur in practice.

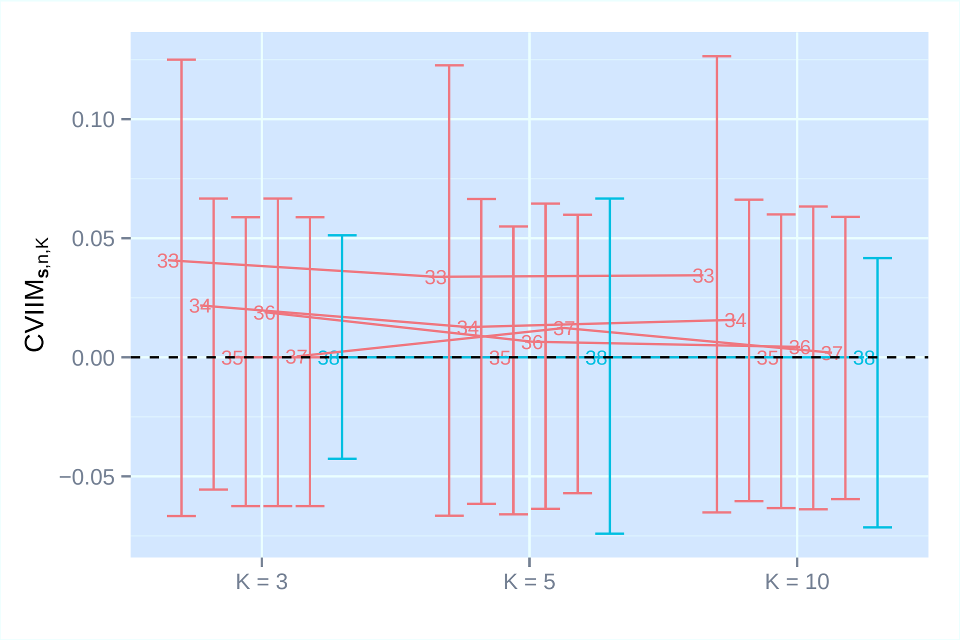

Supplementary Figure 9: $\text{CVIIM}_{\boldsymbol{s},n,K}$-values from imputation study. The numbers distinguish the datasets.

E Dependency of CVIIM on sample size

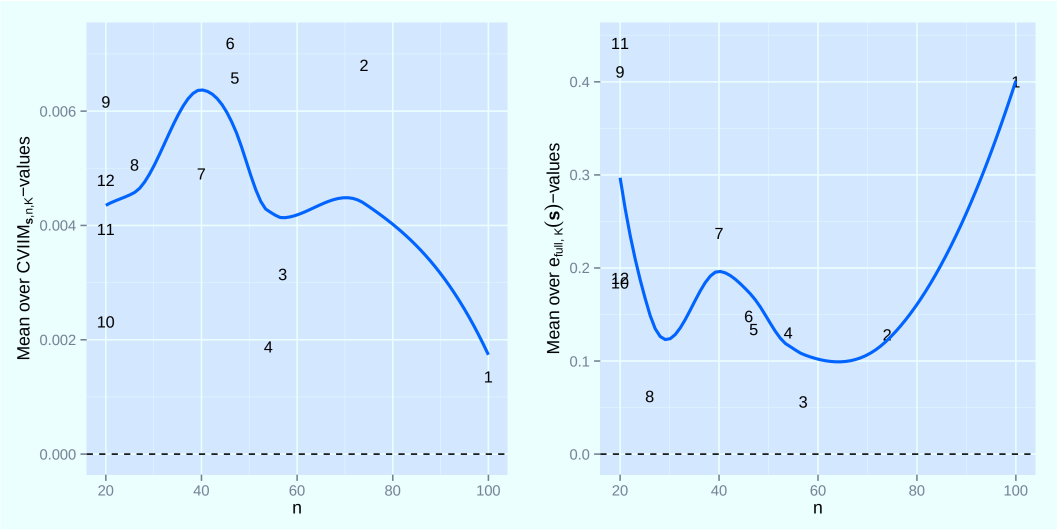

Supplementary Figure 10: Normalization study: Dataset-specific means of the CVIIM$_{\boldsymbol{s},n,K}$- and $e_{full,K}(\boldsymbol{s})$-values plotted against the sample sizes of the datasets. The solid lines are LOESS curves.

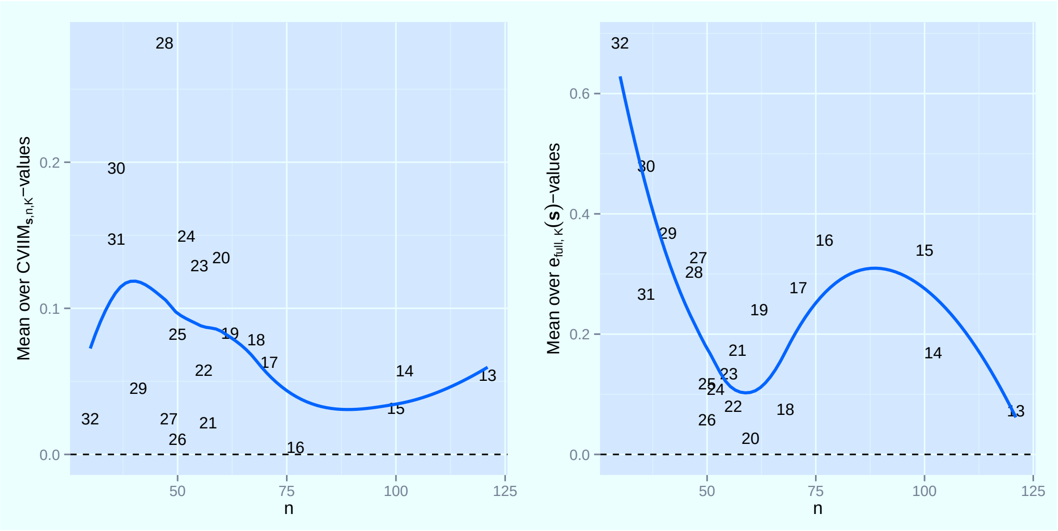

Supplementary Figure 11: PCA study: Dataset-specific means of the CVIIM$_{\boldsymbol{s},n,K}$- and $e_{full,K}(\boldsymbol{s})$-values plotted against the sample sizes of the datasets. The solid lines are LOESS curves.

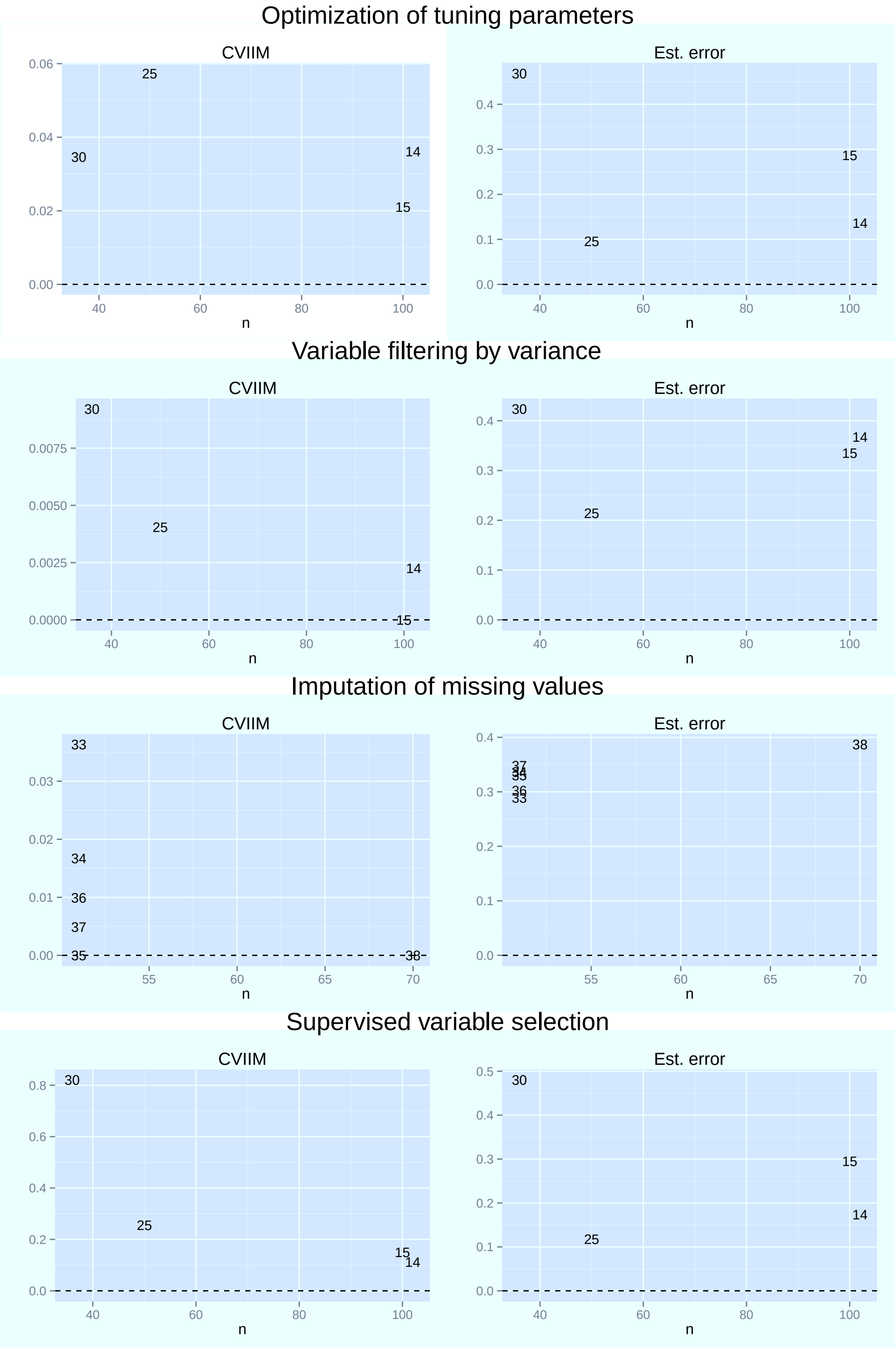

Supplementary Figure 12: Dataset-specific means of the $\text{CVIIM}_{\boldsymbol{s},n,K}$- and $e_{full,K}(\boldsymbol{s})$-values plotted against the sample sizes of the datasets

F Combination of several steps

We considered the following combination of steps: imputation of missing values, supervised variable selection, and optimization of tuning parameters. For imputation the algorithm described in Appendix D was used. For supervised variable selection we chose the 10 variables with the smallest p-values from Wilcoxon's two-sample tests. As a classification method Random Forests with $mtry$ as a tuning parameter was used. Here, $mtry$ was optimized from the grid $\{1, 2, \ldots, 5\}$ through internal CV as described in the section 'Study design' of the main paper.

In addition to $e_{full,K}(s)$ (where all three preparation steps are conducted on the training datasets and addon procedures are used to prepare the test datasets) and $e_{incompl,K}(s)$ (where all three steps are conducted on the whole dataset), for each step we also calculate the "step-specific incomplete CV error" (abbreviation: stspincCVerr) obtained when the considered step is performed using the whole dataset and the others within CV. stspincCVerrs are calculated to investigate the contribution of the individual steps to the discrepancy between $e_{full,K}(s)$ and $e_{incompl,K}(s)$.

In the following we describe the procedures performed to obtain the stspincCVerrs. For the first step (imputation), the stspincCVerr is simply computed by performing the considered step using the whole dataset and then starting the CV repetitions, i.e., all subsequent steps are included in the repeated CV. For steps which are not performed when starting preparation, we have to act differently to obtain a stspincCVerr. In fact, each preceding step changes the data. Therefore we cannot employ ordinary CV in the process of calculating the stspincCVerrs, because it is not possible to perform the considered step only once on the whole dataset. Instead, to obtain a stspincCVerr-value in these cases, we apply the following CV-like procedure using a random partition of the dataset into K equally sized folds as in ordinary CV. For $k \in \{1, \ldots, K\}$: 1) Perform all steps up to the considered step on the k-th training set, each time each time adjusting the corresponding test set with the appropriate addon procedure; 2) Merge training and test set and perform the considered step on the resulting concatenated dataset; 3) Split the result out of 2) into the division of training and test data again; 4) Perform all remaining data preparation steps on the training set, again each time adjusting the test set with the appropriate addon procedure; 5) Calculate the misclassification error of the prediction rule on the test set. We stress that the only goal of this procedure is to assess the impact of the individual steps within the combinations. It has no other meaningful application in error estimation in practice.

As in the analyses of single steps, we also perform $B = 300$ repetitions of the CV(-like) procedures. We used the dataset `ProstatecMethyl`.

The CVIIM$_{\boldsymbol{s},n,K}$-values were 0.2846, 0.3121 and 0.3442 for $K = 3$, $K = 5$ and $K = 10$, respectively. When calculating the step-specific errors (Supplementary Figure 13) it was interesting to notice that when performing only supervised variable selection on the whole dataset the error was virtually identical as when performing all steps on the whole dataset. Correspondingly, incomplete CV based on each of the other steps singly gave results almost no different to full CV. Therefore in this example the supervised variable selection was almost completely alone responsible for the difference between the completely correct and incorrect CV procedure.

We summarize that individual influential steps can play a dominating role when considering combinations of different steps. Note, however, that the analysis presented in this section should

be considered an illustration. The results observed here cannot be generalized, since they can depend strongly on the specific setting and dataset used.

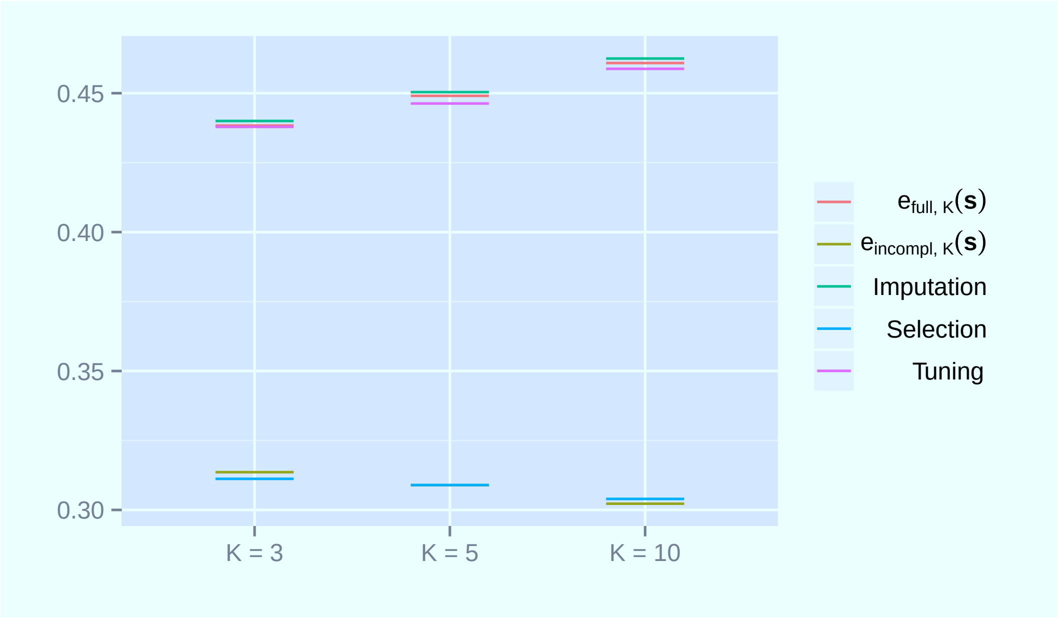

Supplementary Figure 13: Step-specific incomplete CV errors for the data preparation combination of imputation, variable selection and tuning

[1] Tibshirani R, Hastie T, Narasimhan B, Chu G. Diagnosis of multiple cancer types by shrunken centroids of gene expression. Proceedings of the National Academy of Sciences of the United States of America. 2002;99:6567–6572.

[2] Varma S, Simon R. Bias in error estimation when using cross-validation for model selection. BMC Bioinformatics. 2006;7:91.

[3] Bühlmann P, Yu B. Boosting with the L2-loss: Regression and classification. Journal of the American Statistical Association. 2003;98:324–339.

[4] Young-Park M, Hastie T. L1-regularization path algorithm for generalized linear models. Journal of the Royal Statistical Society B. 2007;69:659–577.

[5] Boulesteix AL, Strimmer K. Partial least squares: A versatile tool for the analysis of high-dimensional genomic data. Briefings in Bioinformatics. 2007;8:32–44.

[6] Breiman L. Random forests. Machine Learning. 2001;45:5–32.

[7] Schölkopf B, Smola AJ. Learning with Kernels. Cambridge MA, England: MIT Press; 2002.

[8] Bernau C, Augustin T, Boulesteix AL. Correcting the optimal resampling-based error rate by estimating the error rate of wrapper algorithms. Biometrics. 2013;69:693–702.

[9] Boulesteix AL, Strobl C. Optimal classifier selection and negative bias in error rate estimation: an empirical study on high-dimensional prediction. BMC Medical Research Methodology. 2009;85:9.

[10] Soreq L, Ben-Shaul Y, Israel Z, Bergman H, Soreq H. Meta-analysis of genetic and environmental Parkinson's disease models reveals a common role of mitochondrial protection pathways. Neurobiology of Disease. 2012;45:1018–1030.

[11] Wong J. imputation; 2013. R package version 2.0.1. Available from: `http://CRAN.R-project.org/package=imputation`.
