# Supplementary material for: A measure of the impact of CV incompleteness on prediction error estimation with application to PCA and normalization
Source: BMC Med Res Methodol. 2015 Nov 4;15:95. doi: 10.1186/s12874-015-0088-9 (PMC4634762; doi:10.1186/s12874-015-0088-9)
Supplement: Additional file 1 — Zipped folder containing all necessary R-Code to reproduce and evaluate the real-data analyses and simulations presented in the paper and in Additional file 2 , as well as Rda-files containing all fold errors computed in the real-data analysis enabling fast evaluation of the corresponding results. (ZIP16691 kb) [file 12874_2015_88_MOESM1_ESM.zip › CVIIMCodeData/AddOnFunctionsetc/docval_1.1.6.zip!/docval/doc/doc_by_val.pdf]

Vignette for the documentation by value package (docval)

Dennis Kostka and Rainer Spang

Max Planck Institute for Molecular Genetics
Ihnestrasse 63-73, 14195 Berlin, Germany

Contents

1 Using the software **2**
1.1 Preprocessing 2
1.2 Building the classification rule 2
1.3 Documentation by value 2
1.4 Diagnosing an external patient 3

2 Concepts **3**
2.1 Documenting quantile normalization for add-on preprocessing (rma) . 3
2.2 Documenting the variance stabilizing transformation for add-on preprocessing (vsn) 4
2.3 Documenting an additive model of probeset summary for add-on preprocessing (rma,vsn) 4

1 Using the software

1.1 Preprocessing

We proceed in three steps:

i) Read in the data. We have prepared an `AffyBatch` object called `abo.w`, featuring ten `.cel` files:

```
> library(docval)
> data(willenbrock)

> abo.w

AffyBatch object
size of arrays=448x448 features (27 kb)
cdf=HG-Focus (8793 affyids)
number of samples=10
number of genes=8793
annotation=hgfocus
notes=
```

ii) Preprocessing using `rma`

```
> exs.rma  = wrap.val(abo.w[,-10],method="rma")
> scl.rma  = preproc(description(exs.rma))$val
```

iii) Preprocessing using `vsn`

```
> exs.vsn  = wrap.val(abo.w[,-10],method="vsn")
> scl.vsn  = preproc(description(exs.vsn))$val
```

1.2 Building the classification rule

A classification rule is derived using `pamr`. The function `pamr.fil` is a simple wrapper, performing model selection based on crossvalidation-error.

```
>  labs    = ((as.numeric(pData(abo.w)$IMMUN == "T")-1/2)*2)[-10]
>  sig.vsn =  pamr.fil(exs.vsn,labs,fil=FALSE)
>  sig.rma =  pamr.fil(exs.rma,labs,fil=FALSE)
```

1.3 Documentation by value

We document the classification rules by value: The signature, together with the data-dependent scale information, is stored in a binary format for later application to external samples:

```
> sig.byval.rma = list(sig=sig.rma, scl=scl.rma)
> sig.byval.vsn = list(sig=sig.vsn, scl=scl.vsn)
```

When publishing the signature, the file "sig_byval_rma.rdat" or "sig_byval_vsn.rdat" need to be made available on supplemental web-pages.

1.4 Diagnosing an external patient

We diagnose an external patient (external_patient.CEL.gz). Again, we proceed in three steps:

i) Read in the data. For convenience, we take the left out patient.

```
> abo.extrnl = abo.w[,10]
>
```

ii) Add-on preprocess the data, transforming it to a study-consistent scale. The function wrap.pag.add utilizes the results from the theory section. In order to do that, data-dependent information stored with the signature has to be retrieved.

```
> exs.extrnl.rma = wrap.val.add(abo.extrnl,sig.byval.rma$scl,method="rma")
> exs.extrnl.vsn = wrap.val.add(abo.extrnl,sig.byval.vsn$scl,method="vsn")
```

iii) Predict the class labels of the external patient, using the classifier derived beforehand:

```
> diag.rma = sig.byval.rma$sig(exprs(exs.extrnl.rma))
> diag.vsn = sig.byval.vsn$sig(exprs(exs.extrnl.vsn))
```

2 Concepts

2.1 Documenting quantile normalization for add-on preprocessing (rma)

Assume we have p probes and n arrays. Let $\boldsymbol{X}$ be the $p \times n$ background corrected probe-level expression matrix on log scale. Let $\mathcal{P}$ be a the permutation sorting the columns of $\boldsymbol{X}$ and $\mathcal{P}^{-1}$ its inverse. Then the quantile normalized version $\tilde{\boldsymbol{X}}$ of $\boldsymbol{X}$ is obtained via:

$$\tilde{\boldsymbol{X}} = \mathcal{P}^{-1}((\mathcal{P}\boldsymbol{X})\mathbf{1}) \quad ,$$

where $\mathbf{1}$ is a $n \times p$ matrix with all elements equal to $1/n$. Further on, let $\boldsymbol{\mu}$ be equal to the first column of $(\mathcal{P}\boldsymbol{X})\mathbf{1}$ and let $\boldsymbol{x} \in \mathbb{R}^p$ be an external array. If $\mathcal{P}_{\boldsymbol{x}}$ is the permutation sorting the entries of $\boldsymbol{x}$, the add-on-quantile-normalized version of $\boldsymbol{x}$ consistent with the study is given by via

$$\tilde{\boldsymbol{x}} = \mathcal{P}_{\boldsymbol{x}}^{-1}(\boldsymbol{\mu}) \quad .$$

Since $\mathcal{P}_x$ depends on $\boldsymbol{x}$ only, quantile normalization is fully documented by $\boldsymbol{\mu}$.

2.2 Documenting the variance stabilizing transformation for add-on preprocessing (vsn)

Let $\boldsymbol{X}$ be a raw probe-level $p \times n$ expression matrix of p probes and n samples. The model of Huber et al.[1, 2] relates a random variable X_{ki} ($k = 1 \dots p$ and $i = 1 \dots n$) stochastically with the true abundance μ_k for probe k, given probe k is not differentially expressed:

$$\operatorname{arsinh}(a_i + X_{ki} b_i) =: h_i(X_{ki}) = \mu_k + \epsilon_{ki}, \quad \epsilon_{k_i} \sim N(0, c^2) \quad . \tag{1}$$

Here $a_i \in \mathbf{R}$, $b_i \in \mathbf{R}_+$ and $c \in \mathbf{R}_+$ are unknown parameters. **(author?)** [3] explain how to estimate these parameters, together with μ_k and c from the data at hand. In the main paper, this method is referred to as the `vsn` method.
Assume `vsn` normalized internal data is at hand. That is, for n arrays we have normalized expression values $\{\hat{h}_i(x_{ki})\}$, with $i = 1 \dots n$ and $k = 1 \dots p$, and corresponding parameter estimates $\{(\hat{a}_i, \hat{b}_i)\}$. Further on, a set $\mathcal{K}$ of not differentially expressed genes has been specified. We also have the estimates of the $\hat{\mu}_k$ for each gene $k \in \mathcal{K}$, as well as estimates of the variance of the residuals in Equation (1):

$$\hat{\mu}_k = \frac{1}{n}\sum_{i=1}^{n} \hat{h}_i(x_{ki}) \quad \text{and} \quad \hat{c}^2 = \frac{1}{n|\mathcal{K}|}\sum_{k \in \mathcal{K}}\sum_{i=1}^{n} (\hat{h}_i(x_{ki}) - \hat{\mu}_k)^2 \quad .$$

Given an external $(n+1)$-th sample $\{x_k^\star\}_{k=1}^p$, we want to transform it to the scale determined by the n core arrays. By employing the same stochastic model as for the core data (Equation (1)) and plugging in available estimates, we get the following model for the new sample:

$$\operatorname{arsinh}(a_{n+1} + b_{n+1} X_k^\star) = \hat{\mu}_k + \epsilon_k, \quad \epsilon_k \sim N(0, \hat{c}^2) \quad \text{for} \quad k \in \mathcal{K}.$$

Maximum likelihood estimators for the parameters a_{n+1} and b_{n+1} are available as

$$(\hat{a}_{n+1}, \hat{b}_{n+1}) = \underset{(a,b)}{\operatorname{argmin}} \sum_{k \in \mathcal{K}} \frac{(h(x_k^\star) - \hat{\mu}_k)^2}{2\hat{c}^2} - \sum_{k \in \mathcal{K}} \log(\partial_{x_k^\star} h(x_k^\star))$$

and can be calculated numerically. The estimates are completely determined by the $\hat{\mu}_k$, $\hat{c}^2$ and measurement values on the external array. So is the variance stabilizing transformation $h(x_k^\star) = h(\hat{a}_{n+1} + \hat{b}_{n+1} x_k^\star)$ bringing the $(n+1)$-th sample to the core scale. Therefore `vsn` normalization is fully documented by $\{\hat{\mu}_k\}_{k=1}^{|\mathcal{K}|}$ and $\hat{c}$.

2.3 Documenting an additive model of probeset summary for add-on preprocessing (rma,vsn)

Let $\boldsymbol{X}$ be the $p \times n$ background corrected and normalized probe-level expression matrix on log scale. Let $\boldsymbol{X}^{(k)}$ be the submatrix indexed by the probes belonging to probe set k across all arrays. Then an additive model assumes

$$\boldsymbol{X}_{ij}^{(k)} = p_i + g_j + \epsilon \quad ,$$

where p_i is a probe specific effect and g_j represents the abundance of `mRNA` of gene k in array j. $\hat{p}_i$ and $\hat{g}_j$ can be estimated by a median polish procedure [4]. In that case, $\texttt{median}(\boldsymbol{x}^{(k)} - \hat{\boldsymbol{p}}^{(k)})$ denotes a study-consistent estimate of the expression of gene k, given the external array $\boldsymbol{x}$. $\hat{\boldsymbol{p}}^{(k)}$ is the vector of estimated probe effects associated with probeset k, and $\boldsymbol{x}^{(k)}$ is the vector of normalized expression values of the same probeset. That is, the additive model is fully documented by keeping track of the probe effects $\hat{p}_i$ for all probes i on the array.

References

[1] W Huber, A von Heydebreck, H S/"ultmann, A Poustka and M Vingron. "Parameter estimation for the calibration and variance stabilization of microarray data.", *Statistical Applications in Genetics and Molecular Biology*, 2(1):Art 3, 2003. ISSN 1544–6115.

[2] MA Newton, CM Kendziorski, CS Richmond, FR Blattner and KW Tsui. "On differential variability of expression ratios: improving statistical inference about gene expression changes from microarray data.", *J Comput Biol*, 8(1):37-52, 2001. URL `http://dx.oi.org/10.1089/106652701300099074`.

[3] W Huber, A von Heydebreck, H Sültmann, A Poustka and M Vingron. "Variance stabilization applied to microarray data calibration and to the quantification of differential expression.", *Bioinformatics*, 18 Suppl 1:S96-104, 2002.

[4] JW Tukey. *Exploratory Data Analysis.* Addison-Wesley, 1977.
