# Supplementary material for: A measure of the impact of CV incompleteness on prediction error estimation with application to PCA and normalization
Source: BMC Med Res Methodol. 2015 Nov 4;15:95. doi: 10.1186/s12874-015-0088-9 (PMC4634762; doi:10.1186/s12874-015-0088-9)
Supplement: Additional file 1 — Zipped folder containing all necessary R-Code to reproduce and evaluate the real-data analyses and simulations presented in the paper and in Additional file 2 , as well as Rda-files containing all fold errors computed in the real-data analysis enabling fast evaluation of the corresponding results. (ZIP16691 kb) [file 12874_2015_88_MOESM1_ESM.zip › CVIIMCodeData/AddOnFunctionsetc/docval_1.1.6.zip!/docval/doc/index.html]

R: Vignettes and other documentation

# Vignettes and other documentation

---

## Vignettes from package 'docval'

|  |  |  |  |  |  |
| --- | --- | --- | --- | --- | --- |
| docval::doc\_by\_val |  | Vignette for the documentation by value package (docval) | PDF | source | R code |

## Other files in the doc directory

|  |  |
| --- | --- |
|  | Makefile |
