# Supplementary material for: A measure of the impact of CV incompleteness on prediction error estimation with application to PCA and normalization
Source: BMC Med Res Methodol. 2015 Nov 4;15:95. doi: 10.1186/s12874-015-0088-9 (PMC4634762; doi:10.1186/s12874-015-0088-9)
Supplement: Additional file 1 — Zipped folder containing all necessary R-Code to reproduce and evaluate the real-data analyses and simulations presented in the paper and in Additional file 2 , as well as Rda-files containing all fold errors computed in the real-data analysis enabling fast evaluation of the corresponding results. (ZIP16691 kb) [file 12874_2015_88_MOESM1_ESM.zip › CVIIMCodeData/AddOnFunctionsetc/docval_1.1.6.zip!/docval/html/00Index.html]

R: Documenting microarray preprocessing 'by value'.

# Documenting microarray preprocessing 'by value'.

---

## Documentation for package ‘docval’ version 1.1.6

- DESCRIPTION file.
- User guides, package vignettes and other documentation.

## Help Pages

|  |  |
| --- | --- |
| abo.w | .. data name ... |
| normalize.qnt.add | Add-on quantile normalization |
| normalize.qnt.val | Quantile normalization |
| normalize.vsn.add | Variance stabilizing add-on normalization |
| pamr.fil | .. title |
| summarize.add | Add-on probeset summary |
| summarize.val | Probeset summary |
| vsn.newpars | New variance stabilizing transformation parameters |
| wrap.val | Wrapper for preprocessing, documented "by value" |
| wrap.val.add | Wrapper for add-on preprocessing |
